# Pain Experience in Oncology: A Targeted Literature Review and Development of a Novel Patient-Centric Conceptual Model

**DOI:** 10.3390/cancers17233760

**Published:** 2025-11-25

**Authors:** Chloe Carmichael, Sophie Van Tomme, Jordan Miller, Danielle Burns, Cecile Gousset, Helen Kitchen, Harriet Makin, Natalie V. J. Aldhouse, Paul Cordero

**Affiliations:** 1 Clarivate Analytics, London EC3A 8BE, UKharriet.makin@clarivate.com (H.M.);; 2 Sanofi, 1105 BP Amsterdam, The Netherlands; 3 Sanofi, 94255 Gentilly, France; cecile.gousset@sanofi.com; 4 Sanofi, Reading RG6 1PT, UK

**Keywords:** cancer, pain, patient experience, conceptual model, qualitative research, health-related quality of life

## Abstract

**Simple Summary:**

This review explored pain in cancer patients, including how they describe their pain, and how pain affects their daily lives. The goal was to find better ways to measure pain in cancer treatment studies. Twenty-eight studies covering 534 patients with different types of cancer from various countries were included. The research found that cancer itself and treatments caused pain. Many patients felt pain every day, often describing it as severe, long-lasting, and “sharp,” “stabbing,” or “shooting.” Pain greatly affected patients’ emotions, daily activities, sleep, work, and physical and social wellbeing. Patients struggled to talk about their pain needs with healthcare providers and many feared using opioid pain medications. A visual overview was created that explains the patient experience of cancer-related pain. This visual guide can help patients and doctors talk more effectively about pain and can help researchers develop better ways to measure pain in cancer treatment studies.

**Abstract:**

Background and objective: Typical endpoints in cancer clinical trials focus on standardized efficacy endpoints, such as overall survival. Pain is not always assessed, although it is a highly prevalent and distressing aspect of patients’ cancer experience and plays a critical role in health-related quality of life. To inform future pain measurement strategies in oncology, this targeted literature review of pain-related qualitative publications in oncology aimed to characterize and explore the patient experience of pain, and its impact on how patients feel and function. Methods: A review of publications in MEDLINE, Embase and PsycINFO from 2018 to 2023 was conducted. Patient quotes or author descriptions/interpretations were extracted and analyzed with directed content analysis techniques, using ATLAS.ti v9. Data were synthesized to inform the development of a conceptual model. Results: Twenty-eight publications, with data from 534 patients across different oncology indications and geographies, were reviewed. Pain was triggered by disease symptoms and treatment, including surgical procedures, chemotherapy, and radiation. Pain was most often daily, severe, and chronic in nature. Characterizations of pain varied, but most often “sharp”/“stabbing”/“shooting” pain was described across different treatment stages. Pain had an extensive impact on emotional wellbeing, activities of daily living, physical, physiological and social functioning, sleep and work. Unmet needs included difficulty communicating pain needs to healthcare practitioners and fear/distrust of opioid pain medication. Conclusions: This research provides a patient-centric model conceptualizing the patient experience of cancer-related pain. The findings highlight the burden and all-encompassing impact of cancer-related pain, demonstrating the importance of assessing pain in oncology clinical trials.

## 1. Introduction

Pain, including nociceptive and neuropathic pain, is a common symptom in many cancers [1], occurring in 45% of adult cancer patients globally [2]. Approximately a third of patients experience moderate to severe pain [2], and pain remains poorly controlled in around 40% of patients [3]. Improvements in survival due to advances in cancer treatments may be associated with extended chronic pain [4]; although the prevalence of pain is highest in advanced, metastatic, and terminal cancer patients (55% of patients), a substantial proportion of patients report pain after curative treatment (36% of patients), with approximately a quarter of these patients experiencing moderate to severe pain [2]. Although pain is not immediately life-threatening, previous studies indicate that the presence of pain is associated with poorer prognosis in cancer [5], along with impaired functioning, wellbeing, and quality of life [6,7,8]. Studies report a need for more effective pain treatments, and for those pain treatments to be personalized to each patient’s characteristics, preferences, and pain experience [9,10].

Identifying important pain experience-related concepts of interest, such as the signs, symptoms, and impacts of cancer-related pain from the patient perspective, is essential for achieving patient-focused drug development (PFDD). This understanding can then inform the selection of fit-for-purpose clinical outcome assessments to measure those concepts of interest in clinical trials [11]. Conceptualizing the patient experience, typically through the development of a conceptual model, is a key initial step in identifying clinical outcome assessments (COAs), such as patient-reported outcomes (PROs) to measure pain. Though existing models and visual representations provide insights into the experience of pain in oncology, they are limited by sample size [12] or are indication-specific [13]. They also do not provide a comprehensive, holistic overview of pain across oncological indications, or explore how pain may be linked to functional and health-related quality of life (HRQoL) impacts.

The primary objective of this study was to characterize and explore the patient experience of cancer pain symptoms and the associated impact on how patients feel and function, including aspects of HRQoL, by using a targeted qualitative literature review. The study also aimed to identify any existing conceptual models depicting the patient experience of cancer-related pain.

## 2. Materials and Methods

### 2.1. Eligibility Criteria

Studies with a qualitative methodology that describes the patient experience of cancer-related pain, across all oncology indications, published in English between January 2018 and August 2023, were eligible for inclusion. This timeframe was selected to ensure a contemporary representation of cancer pain, in acknowledgment that advances in oncology treatment and management may have influenced the patient experience. Full details of the eligibility criteria are detailed in Table 1.

### 2.2. Data Sources, Search Strategy, and Quality Assessment

Relevant articles (full publications or conference abstracts) indexed on MEDLINE^®^, Embase, and PsycINFO (all on Ovid) were identified using database search strings. Three search strings were developed to account for differences in syntax within each database and combined terms for “cancer” AND “pain” AND “qualitative research studies” OR “conceptual models”. Supplementary hand-searching of the Food and Drug Administration (FDA) and European Medicines Agency (EMA) websites, and the reference lists of any included studies identified from database searches, was conducted (Supplementary Material File S1).

**Figure 1:**
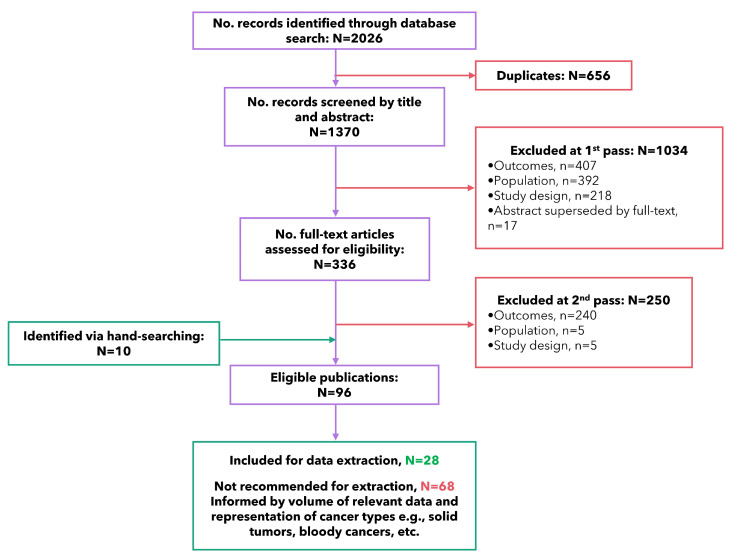
PRISMA diagram.

Electronic database searching was conducted on 21 August 2023. All identified records were downloaded into a bespoke Microsoft Access database used to manage citation screening. Following deduplication, studies were initially screened by their titles and abstracts (first pass) by two authors. Potentially relevant studies were obtained in full and reviewed (second pass) by two authors. Any discrepancies were resolved through discussion with the wider team of authors. The reasons for exclusions at the first and second pass (in accordance with the eligibility criteria presented in Table 1) were summarized in a preferred reporting items for systematic reviews and meta-analyses (PRISMA) flow-chart, based on the 2009 guidelines (Figure 1). The quality of the included studies was assessed by one author, using the Critical Appraisal Skills Programme (CASP) checklist for qualitative studies (Table S1) [14].

### 2.3. Data Extraction and Analysis

Data extraction and analysis of patient quotes or author descriptions/interpretations was conducted by two authors who were trained in qualitative research techniques. The extracted data underwent secondary analysis using content analysis for concept elicitation [15], based on semantic, qualitative, directed content analysis techniques, using a combination of inductive and deductive coding [16,17]. The analysis largely followed an experiential, realist approach, which focused on participants’ individual perspectives and experiences.

The following five-step process of qualitative directed content analysis was applied [15]: immersion in the data, coding, iterative review of codes, defining and refining concepts and domains, and reporting. Coding of extracted data was facilitated by the qualitative analysis software, ATLAS.ti 9.1.7.0 (Atlas.ti Scientific Software Development GmbH, Berlin, Germany). A conceptual model was developed from the coded data to present a visual illustration of the experience of cancer-related pain.

## 3. Results

### 3.1. Study Selection

The electronic search yielded 2026 records. After duplicates (*n* = 656) were removed, records were screened by title and abstract (first pass, *n* = 1370). Records which did not meet the eligibility criteria were removed (*n* = 1034), and the full-text publications of the remaining records were screened (second pass, *n* = 336). Upon review of the full publications, 96 publications appeared to be relevant for inclusion and were eligible for data extraction (*n* = 250 excluded, *n* = 10 identified via supplementary hand-searching from FDA/EMA webpages and references from included studies).

To select the most relevant publications for the development of the conceptual model, a study selection meeting was held with all authors. Publications were initially ordered on the volume of extractable qualitative data (direct quotes or author interpretations), pertaining to the review objective to understand the patient experience of cancer-related pain and the associated impacts on HRQoL. Papers were prioritized to ensure varied representation of cancer types (e.g., solid tumors, blood cancers, etc.) and varied representation of the stages of the cancer journey. Any qualitative publications containing a conceptual model of pain in oncology were also recommended for inclusion. The 28 most relevant publications were retained for data extraction (Figure 1). A list of eligible publications that were not retained for data extraction during screening are provided in Table S3.

### 3.2. Study Characteristics

Across the 28 included studies, the experiences of 534 patients with cancer-related pain were reported (Table 2).

**Figure 2:**
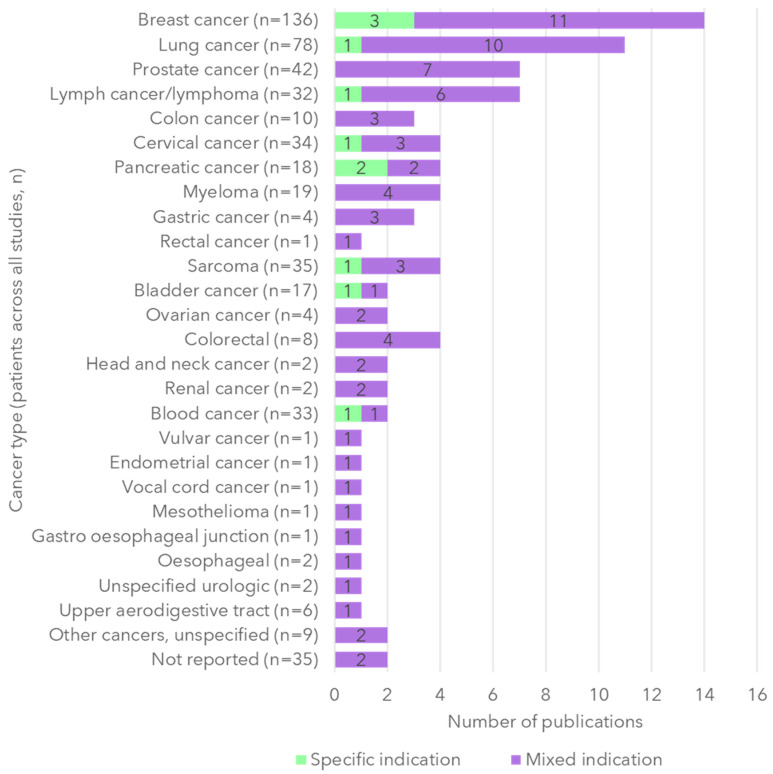
Summary of study oncology indications.

The most frequently identified indications were breast cancer [18,19,23,26,27,30,34,35,36,37,38,41,42,43] and lung cancer [18,25,27,30,34,35,37,39,41,42,43], with 15 studies, including a mixed population of cancer indications [12,18,19,20,23,25,27,30,33,34,35,37,41,42,43] (Figure 2). Studies were most frequently conducted within Europe [12,18,19,20,22,23,24,26,29,37] and the United States [22,28,31,32,33,34,35,38,40,42,43], and utilized semi-structured interviews as a method of data collection [12,13,18,19,20,21,22,24,25,26,27,29,30,31,32,33,34,36,37,38,39,40,41,42,43]. A variety of analytic approaches were used: most often, thematic analysis [13,18,21,29,30,34,37,38] and content analysis [23,24,26,27,40,42,43]. Details of the methodology and analytic approach applied by each of the included studies are available in Table S2.

The number of studies mentioning each concept evaluated (pain triggers, pain descriptors, pain impacts, coping strategies, and unmet needs) and supportive quotes are presented in the supplementary tables.

### 3.3. Pain Triggers

The most frequently reported triggers for patient pain experiences included procedures, such as bone marrow biopsies or tumor removal surgery [13,21,24,26,27,28,29,35,36,37,39,41], and treatment, including chemotherapy, hormone therapy, and radiation [12,13,22,32,35,36,38,41,42]. Pain was also commonly triggered or caused by the cancer itself, including primary tumors, tumor growth or cancer metastasis, particularly by bone metastases [13,25,27,32,35,37,42].

Daily activities triggered pain, such as walking [18,20,30] or movement, including general pain from everyday movements, acute pain when mobilizing following tumor removal surgery, or longer-term pain when reaching or moving the arms following breast cancer surgery [12,20,24,26,39,41]. Passing urine or stool also triggered pain [32,35,43].

For some patients, pain was triggered by the time of day and tended to be worse at night [12,22,27,39,42], or exacerbated by negative thinking, including stress or worrying [27,35,41]. Less commonly reported triggers included eating [29,30], the weather (both cold and warm) [22,26], sneezing or coughing [22,42], physical activity or overexertion [18,35], or inactivity and sedentary behavior [35,38] (Table S4).

### 3.4. Pain Descriptors

Pain descriptors were stratified into temporal stages according to whether the pain was experienced: (1) pre-treatment/diagnosis; (2) while living with cancer or during active treatment, (3) post-treatment; or (4) in cancer survivors. Most pain was experienced while undergoing active treatment or living with cancer, although the descriptors used to characterize pain experiences varied substantially; sharp/stabbing/shooting pain was the only pain descriptor reported across all four temporal stages, including patients with solid tumors and hematologic malignancies (Table S5).

The frequency of cancer-related pain was not commonly described, and it varied across the temporal treatment stages. Most publications reporting on pain frequency noted that pain was either a daily [26,32,34,38,43] or intermittent occurrence [29,30,32,38]. These publications largely reported on patients living with cancer or during active treatment [29,30,32,38], or pain experienced post-treatment [26,32,34,38,43] and by survivors [26,32,34,38,43]. For other patients living with cancer or during active treatment, pain was described as a persistent occurrence “that never subsides” [27,32,35], and the frequency was reported to be unpredictable [18,30] (Table S6).

The most commonly reported duration of pain was chronic, enduring pain that was “always there” (*n* = 10 publications, *n* = 7 of which reported on patients living with cancer or during active treatment and *n* = 3 of which reported on patients post-treatment or survivors) [19,22,27,28,31,34,35,36,41,43]. Other studies described pain as being short-lived [23,29], lasting either days [21,22] or weeks [22,37] at a time, or varying in duration [35,38] (Table S7).

Throughout most publications, and across all temporal treatment stages, cancer pain was reported to be severe [18,19,20,21,22,23,24,25,26,27,29,30,31,32,33,34,35,36,38,39,40,41,42,43]. Many patients described their pain as unbearable [19,20,21,24,25,27,30,31,32,33,35,36,39,41,42,43], often using powerful language such as “terrible”, “excruciating”, “overwhelming” or “torture”. Within five publications describing patient experiences while living with cancer and receiving active treatment, patients recounted either a desire to die or expressed death-related thoughts when detailing their experience with unbearable pain [27,30,31,41,42]. Less frequently, publications reported that the severity of pain was variable [18,19,22,24,28,32,35] or mild [12,22,25,36,38,39] (Table S8).

**Figure 3:**
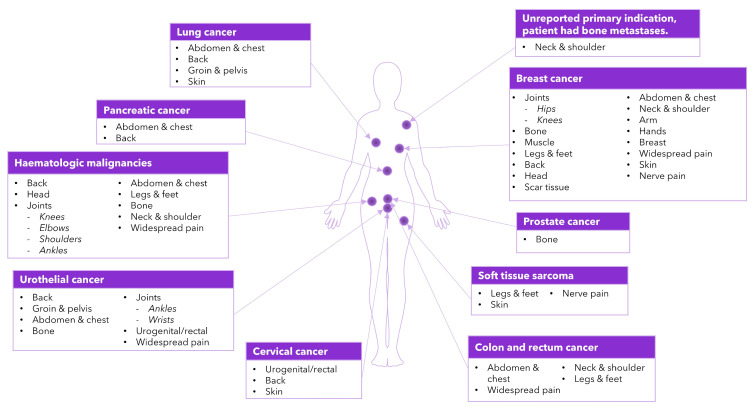
Body map of pain locations by oncology indication, as reported across the qualitative literature.

Pain occurred across multiple areas of the body, due to primary tumor sites, disease progression or metastasis, and the side effects of treatment (Figure 3). Pain was most often experienced in the back [12,21,22,25,27,32,35,37,42], abdomen or chest [20,22,25,27,29,30,32,35,39], across multiple widespread locations [19,22,27,28,30,34,35,38,42], joints [22,31,35,36,37,38,40,42], bones [12,18,22,32,35,38,41,42], legs and feet [22,30,35,38,40,42], head [22,25,27,35,38,40], or knees [22,35,36,40,42] (Table S9).

### 3.5. Pain Impacts

#### 3.5.1. Physiological

Frequent physiological impacts of cancer-related pain included fatigue [19,25,28,30,31,32], weakness [25,27,30,38,41], or a sense of exhaustion [28,31], which could occur following episodes of severe or breakthrough pain, or be attributed to pain-related sleep disturbances, the side effects of treatment, or cancer recurrence. Pain also reduced patients’ appetite [25,29,39,41]. Less commonly described physiological impacts included sweating or shivering [12,30], itching [12,26], breathing difficulties [20,25], and dizziness [25,30] in response to pain (Table S10).

#### 3.5.2. Physical

Pain had a substantial impact on aspects of physical functioning, most frequently in relation to walking; patients were often either unable to walk or walk without assistance [20,22,25,30,32,34,39,40,41,42,43], or experienced a reduction in their ability to engage in exercise [18,20,23,26,30,32,34,41,42,43]. For some patients, pain impacted their physical ability and motivation to leave their bed [25,27,41], or caused significant restrictions on basic movement and left them immobile during episodes of severe or breakthrough pain [31,41,43]. Others described being unable to sit down comfortably due to pain [25,27,30], challenges with transitional movements [30], or a limited range of motion causing restrictions in bending or in free movements in the arms following breast cancer treatment [26,31,33,41] (Table S11).

#### 3.5.3. Emotional

Across the included studies, cancer-related pain had numerous pervasive impacts on HRQoL, including patient emotional wellbeing. Patients frequently reported fear, particularly in anticipation of upcoming treatment or unpredictable breakthrough pain, or that pain signified a worsening or progression of their disease [12,18,19,21,22,23,24,26,28,29,30,37,38,41,42], with pain often leading to anxiety/worry [19,21,22,24,25,30,34,36,42]. Pain commonly resulted in feelings of sadness/depression [20,25,26,27,30,34,38,41,42] and a sense of hopelessness [25,28,30,31,33,38,41,42], with some patients describing fatalistic beliefs that their cancer pain was inevitable [20,25,30,33] and a sense of isolation [25,28,37,41,43]. Anger/frustration [25,26,27,30,34,38,41,42] or irritability [25,27,38,41] was also frequently described. Severe or unbearable pain, or lack of pain control, led to some patients reporting distress [29,31,34,43] and suicidal or death-related thoughts [27,30,31,41,42]. Pain induced feelings of helplessness [20,21,24,36,38], a loss of self/autonomy [20,26,27,34,43], and feelings of embarrassment or shame due to an increase in care requirements, because of severe pain [20,23,27,29]. For some patients, low self-esteem or a sense of worthlessness was described [27,41]. However, a sense of resilience or inner strength when coping with pain was noted as a positive emotional impact [20,21,30] (Table S12).

#### 3.5.4. Activities of Daily Living (ADLs)

Cancer-related pain substantially impacted ADLs for patients, including the ability to complete daily household tasks/chores [19,25,26,31,34,35,41,42] or engage in previously enjoyable leisure activities or hobbies [18,20,26,32,34,35,41,42], which might lead to patients reporting a reduction in their sense of independence [20,25,27,30,31,42]. Pain also impacted patients’ self-care tasks, such as bathing and dressing [20,31,40,41] and eating [27,30,34,42]. Less frequently reported impacts included difficulty caring for dependent others [22,27] and an increase in staying at home due to pain [20,37] (Table S13).

#### 3.5.5. Sleep

Duration of sleep [12,25,30,31,37,41,42], sleep quality, and efficiency [25,30,37,38,39] were negatively impacted by pain, with disrupted low-quality sleep leading to irritability [38] and a sense of hopelessness [25]. Challenges with sleep initiation were also reported; patients were unable to fall asleep due to severe pain, or until medication had reduced the pain (Table S14) [25,30,33,39,42].

#### 3.5.6. Social Functioning and Relationships

Cancer-related pain restricted patients’ involvement in social events by reducing their ability and motivation to travel to or engage in social activities, leading to isolation [20,25,26,32,43]. Pain also negatively impacted patients’ roles within their families or relationships with family members, with patients often struggling with the impact of cancer and cancer pain on their children, reporting shame in being physically unable to contribute to family life, and requiring increased support from their children or other family members [27,32,41]. Pain adversely impacted romantic relationships, with a loss of independence and increased physical caring requirements influencing relationship dynamics and intimacy (Table S15) [20,32].

#### 3.5.7. Work and Financial

Cancer-related pain caused disruptions to working life, due to time off or reduced functioning, and performance, because of pain and associated fatigue [25,26,34,38,41,43]. Some patients were unable to work in any capacity due to pain [27,34,41,42,43], and loss of earnings was described as a financial consequence of pain-related disruptions to working life (Table S16) [25,27].

### 3.6. Coping Strategies

The most frequently described strategy to alleviate or cope with cancer-related pain was the use of medication: primarily pain medication [12,18,19,21,23,24,25,27,29,30,31,32,33,35,36,38,39,41,43] or antidepressants [25,36,38]. Patients also utilized specific self-management strategies, such as keeping medication easily accessible or timing medication to avoid breakthrough pain [12,18,19,20,23,31,33,38,43], and developed their knowledge of the causes and triggers of pain, the appropriate use of pain medication, and of the disease and treatment [20,23,31,33,36]. A change or break in treatment was used as a coping strategy in those receiving adjuvant hormonal therapy for breast cancer [36,38].

Lifestyle strategies were also described, including light physical activity such as walking [12,20,25,36,38,41], positional changes and stretching [12,25,29,30,35,36,38,41], or dietary changes [27,30,36]. In addition, warm baths, heat packs, or ice packs were used [19,25,29,30,33,36]. Less commonly, patients reported using sleep as a coping strategy [19,25].

Psychological strategies included practicing resilience and stoicism [12,18,20,23,25,27,30,31,33,34,36,38,43], spirituality and prayer [20,21,25,27,31,33,41,43], optimism and positivity [20,21,27,29,34,38], concealing pain from others [12,18,19,20,29,30,31,34,36,43], and distraction [18,20,25,27,41]. Uses of massage [27,29,30,33,36,38,42] and alternative medicine were also reported, including acupuncture and bloodletting [23,25,27,30,33,36,38].

Patients accessed various support networks to provide physical and emotional support during their experience with cancer-related pain, such as healthcare professionals [19,20,24,31,33,38], family members and partners [18,20,33,38,40,43], or wider networks of friends and other cancer patients via online forums [18,20,27,36,38]. Hospice staff/services or dedicated pain clinics were also used (Table S17) [19,25,36].

### 3.7. Unmet Needs

Patients frequently described concerns around communicating their pain to their healthcare providers [18,19,20,23,24,25,27,30,34,36,38,43] and about the efficacy of the available pain medication [19,21,24,27,29,30,33,36,39,41,43], noting that their medication was ineffective for managing severe pain. Studies frequently reported that patients described negative attitudes or fears around opioids: namely, a fear of addiction [12,23,29,30,33,35,36,41,43]. Concerns about the side effects of pain medication, such as constipation and drowsiness/dizziness, were also frequently reported, often in relation to opioids [18,25,30,33,35,41,42].

Other common unmet treatment needs included a lack of information and knowledge related to cancer treatment and pain [20,23,24,29,30,36,37,38], timely/easy access to pain medication [24,25,27,30,31,33,43], and options for pain management [27,30,34,38], as well as challenges with accessing healthcare professionals or providers in relation to pain [18,31,34] (Table S18).

### 3.8. Conceptual Model of the Patient Experience of Pain in Oncology

**Figure 4:**
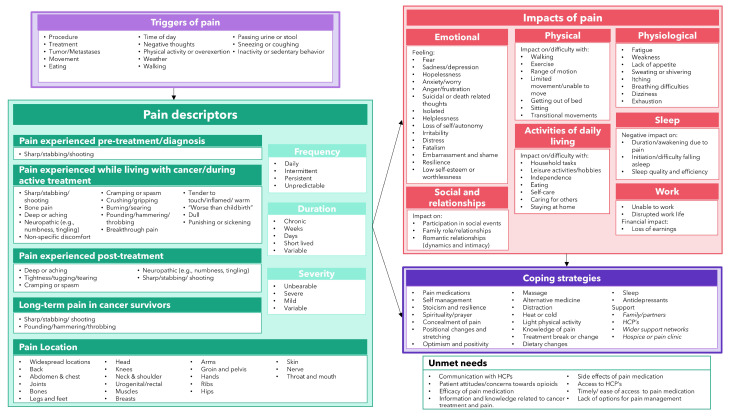
Conceptual model of the patient experience of pain in oncology.

Though models of cancer-related pain in soft tissue sarcomas [13] and in solid tumors [12] were found during the searches, a comprehensive holistic conceptual model depicting the patient experience of cancer-related pain was not identified. A conceptual model was therefore developed (Figure 4). The model provides a visual depiction of pain triggers, pain descriptors across temporal cancer stages, and insights into the frequency, duration, and severity of pain, the impact of pain on patient HRQoL, coping strategies, and patients’ unmet needs.

## 4. Discussion

This review of 28 primary qualitative studies (*n* = 534 patients across a variety of countries and oncology indications) identified and synthesized over 100 unique concepts, which were aggregated into pain triggers, descriptors, frequency, severity, duration, and impact on HRQoL domains, to conceptualize the patient experience of cancer-related pain. Pain affected individuals with cancer across all disease stages and could be triggered by cancer itself or by treatment. Pain frequency varied from daily or intermittent to ‘never subsiding’ and was unpredictable for some patients. Similarly, the duration of pain differed; both acute and chronic, enduring pain that was ‘always there’ were reported. In most publications, cancer-related pain was described as being severe or unbearable, and substantially impacted patients’ emotional and physiological wellbeing, ability to complete ADLs and maintain their independence, physical and social functioning, sleep, work, and finances. Several clear unmet needs were identified: patients reported difficulty communicating their pain needs to healthcare practitioners and a considerable distrust of opioid pain medication due to fear of addiction. The all-encompassing nature of pain and its impact across all aspects of patients’ lives highlights the importance of assessing the intensity of pain and its impact on HRQoL in oncology clinical trials, in addition to other key oncology symptoms and traditional endpoints (e.g., survival and time to progression).

Conceptual models can be used to represent the health experiences of patients, descriptors of those experiences, and the concepts of interest for assessment in clinical trials, helping drug developers to communicate the concept to be measured and regulatory reviewers to determine whether a proposed COA/PRO captures the entirety of a concept of interest [11]. Our findings informed the development of a new, holistic conceptual model of pain in oncology. This model built upon the high-level concepts identified by Barrett et al., 2023, who focused on pain and fatigue in soft tissue sarcoma and conceptualized pain across nine concepts within two broad domains (“pain descriptors” and “pain impact”) [13], and the concepts identified by Restivo et al., 2023, who developed a grounded theory model, conceptualizing pain representation and coping strategies based on a small sample of adults with solid tumors [12]. The conceptual model developed in this study provides an important basis for selecting, developing, or modifying PRO measures to assess relevant pain outcomes in oncology clinical trials, which also has important applications for routine clinical practice; integrating PROs into clinical practice may enhance patient-centered care and facilitate improved patient and clinician discussions about pain [44]. Moreover, where appropriate, cancer patients should be referred to dedicated pain management and palliative care units to ensure comprehensive support. The importance of pain in oncology is well established, but no standardized, accepted classification system for cancer pain currently exists [10]. As patient survival improves with the development of more efficacious treatments, the assessment of concepts related to HRQoL, including measuring pain using PROs, is critical for PFDD, which is encouraged by regulatory bodies [11]. This review illustrates that the measurement of pain, as a disease-related symptom, is pertinent to include as part of a core PRO strategy in oncology clinical trials [45]. The involvement of palliative care clinical units should also be considered in the design of clinical trials, to provide guidance on pain assessment and monitoring. As the experience of pain is influenced by the tumor type and location, as well as treatment, and may be of greater importance to patients with certain types of cancers than with others, the conceptual model should be applied through a disease- and population-specific lens to ensure that the concepts that are most relevant to the context of use are assessed.

Several PRO measures exist in oncology that assess aspects of pain to varying degrees of comprehensiveness. Future research should explore the available PRO measures that may be suitable for use in different oncology clinical research scenarios, considering the context of use and relevant industry guidelines (such as the Initiative on Methods Measurement and Pain Assessment in Clinical Trials recommendations [46] and the FDA PFDD Guidance [47]), and should focus on understanding the optimal timing for measuring symptoms, to best utilize the PROs measures [48].

This review encompassed many patients over a wide range of countries and oncology indications to inform the development of a novel conceptual model. To our knowledge, this model is the first developed for cancer-related pain that combines insights from individuals living with different oncological diagnoses and at different disease stages (from active treatment to cancer survivors). As such, this model provides important insights into the concepts that are relevant to assess in oncology clinical trials, as well as describing coping strategies and unmet needs. However, many of the published qualitative studies included mixed populations of cancer indications and did not clearly distinguish between indications when providing participant quotes. Formal sub-group analysis was not included in this literature review; therefore, it is difficult to determine whether the experience of pain is comparable across all forms of cancer. Previous research has demonstrated discrepancies in pain perception between men and women [49]. Sex or gender-based analyses were not included in the scope of this review; however, future research could seek to elucidate whether these differences persist across the pain experienced in various types of cancer.

## 5. Conclusions

This review informed the development of the first patient-centric conceptual model of the experience of cancer-related pain across oncological indications, disease stages, and treatments. The findings demonstrate that pain is a critical, impactful symptom for patients in many cancer indications, and that patients experience clear unmet needs with regard to pain management, including difficulty communicating their pain needs, concerns around the efficacy of pain medication, and distrust of opioid pain medication. Therefore, pain needs to be assessed appropriately in future oncology clinical trials. Future research should explore which PROs and COAs are used in oncology clinical trials and review how key stakeholders understand, recommend, and include pain in their assessments.

## Figures and Tables

**Table 1:** Eligibility criteria.

Criteria	Include	Exclude
Population	- Adults diagnosed with any type of cancer	- Studies which enrolled pediatric participants only - Studies with mixed populations were eligible if data were reported separately for adults and pediatrics
Intervention/ Comparator	- No restrictions	- No restrictions
Study design	- Studies with qualitative methodology (e.g., qualitative interviews or focus groups)	- Studies with quantitative methodology only - Case reports - Review papers were not eligible for inclusion, but highly relevant review papers (i.e., those summarizing qualitative research) were reviewed to determine whether any of the reviewed primary studies were eligible for inclusion via handsearching methods
Outcomes	- Patient experience of cancer-related pain - Patient descriptions of patient’s cancer pain and associated health-related quality of life impacts - Outcomes which were reported as direct quotes or author summaries of collected data	- Patient experience of other symptoms and impacts of cancer or cancer treatments (i.e., not pain) - Quantitative results only (e.g., COA measure scores) - Studies using multiple-choice survey methodologies were flagged for discussion and highly relevant publications were included if there was a lack of fully qualitative research
Publication language	- English - Non-English publications with English titles and abstracts	- Non-English publications with non-English titles/abstracts
Date of publication	- Published between January 2018 and date of searches (21 August 2023)	- Published prior to January 2018

Abbreviations: COA, clinical outcomes assessment.

**Table 2:** Characteristics of included studies (*n* = 28).

Study	Sample Size, *n*	Cancer Type(s)	Females, *n*	Age, Years (Mean [Range])	Country
Adam, 2018 [18]	14	Mixed population (breast, *n* = 1; lymphoma, *n* = 1; renal/pancreatic, *n* = 1; prostate, *n* = 2; colorectal, *n* = 2; ovarian, *n* = 1; lung, *n* = 3; gastro-esophageal junction, *n* = 1; esophagus, *n* = 2)	4	Not reported [56–76]	GB
Allsop, 2019 [19]	13	Mixed population (prostate, *n* = 7; breast, *n* = 3; head and neck, *n* = 1; colon, *n* = 1; mesothelioma, *n* = 1)	5	67.77 [50–83]	GB
Appleyard, 2018 [20]	8	Mixed population (myeloma, *n* = 3; prostate, *n* = 3; colorectal, *n* = 1; bladder, *n* = 1)	1	76.38 [72–85]	GB
Barrett, 2023 [13]	28	Soft tissue sarcoma	22	43 [22–79]	Not reported
Benali, 2022 [21]	31	Cervical	31	Not reported [27–70]	Morocco
Cella, 2023 [22]	30	Acute myeloid leukemia	17	Not reported [28–72]	US, Germany
Ekstedt, 2019 [23]	20	Mixed population (breast, *n* = 7; prostate, *n* = 9; other [not specified], *n* = 4)	8	Not reported [45–83]	Norway
Englid, 2023 [24]	12	Pancreatic	5	67 [56–74]	Sweden
Erol, 2018 [25]	16	Mixed population (lung, *n* = 8; colon, *n* = 6; gastric: *n* = 2)	3	62.75 [not reported]	Turkey
Everaars, 2021 [26]	26	Breast	26	56.9 [32–77]	The Netherlands
Hassankhani, 2023 [27]	17	Mixed population (breast, *n* = 5; colon, *n* = 3; lymphoma, *n* = 3; blood, *n* = 3; lung, *n* = 1; gastric, *n* = 1; vulvar, *n* = 1)	12	39.4 [21–55]	Iran
Hodge, 2022 [28]	17	Not reported	16	Not reported	US
Koulouris, 2021 [29]	4	Pancreatic	2	Not reported [61–82]	GB
Liu, 2018 [30]	9	Mixed population (lung, *n* = 1; breast, *n* = 2; pancreatic, *n* = 1; gastric, *n* = 1; cervical, *n* = 1; prostate, *n* = 1; rectal, *n* = 1; lymph, *n* = 1)	4	Not reported [37–76]	China
Maly, 2018 [31]	18	Not reported	Not reported	Not reported	US
Martin, 2022 [32]	16	Bladder	8	53.8 [46–64]	US
Nabulsi, 2023 [33]	20	Mixed population (multiple myeloma, *n* = 10; leukemia, *n* = 5; lymphoma, *n* = 4; myelofibrosis, *n* = 1)	12	Not reported [not reported]	US
O’Regan, 2022 [34]	13	Mixed population (breast, *n* = 9; lung, *n* = 3; head and neck, *n* = 1)	10	57 [40–81]	US
Restivo, 2023 [12]	16	Mixed population (upper aerodigestive tract, *n* = 6; sarcoma, *n* = 5; gynecologic, *n* = 3; urologic, *n* = 2)	8	56.18 [22–82]	France
Schumacher, 2021 [35]	42	Mixed population (prostate, *n* = 17; breast, *n* = 14; lung, *n* = 6; other unspecified, *n* = 5)	17	64.0 [not reported]	US
Smith, 2023 [36]	14	Breast	14	55.8 [not reported]	Australia
Vestergaard, 2023 [37]	7	Mixed population (breast, *n* = 3; lung, *n* = 3; endometrial, *n* = 1)	6	66.43 [not reported]	Denmark
Walsh, 2022 [38]	30	Breast	30	55.13 [27–76]	US
Wei, 2022 [39]	39	Lung	24	Not reported [42–82]	China
Whisenant, 2021 [40]	21	B-Cell lymphoid malignancies	5	61.4 [not reported]	US
Xu, 2019 [41]	12	Mixed population (lung, *n* = 4; breast, *n* = 3; colorectal cancer, *n* = 2; myeloma, *n* = 1; liposarcoma, *n* = 1; non-Hodgkin’s lymphoma, *n* = 1)	6	Not reported	China
Yeager, 2018 [42]	27	Mixed population (breast, *n* = 9; lung, *n* = 8; prostate, *n* = 3; ovarian, *n* = 3; cervical, *n* = 1; leiomyosarcoma, *n* = 1; renal cell, *n* = 1; vocal cord, *n* = 1)	18	57 [30–79]	US
Yeager, 2023 [43]	14	Mixed population (multiple myeloma, *n* = 5; colorectal, *n* = 3; lung, *n* = 2; breast, *n* = 2; cervical, *n* = 1; lymphoma, *n* = 1)	9	Not reported [not reported]	US

Abbreviations: GB, Great Britain and US, United States.

## Supplementary Materials

The following supporting information can be downloaded at: https://www.mdpi.com/article/10.3390/cancers17233760/s1, Table S1. Stage 1: CASP quality assessment of included studies. Table S2. Methodology of the included studies. Table S3. Eligible publications that were not retained for data extraction. Table S4. Pain triggers reported in the qualitative literature. Table S5. Pain descriptors across treatment stages. Table S6. Frequency of pain experienced in the qualitative literature. Table S7. Duration of pain experienced in the qualitative literature. Table S8. Severity of pain experienced in the qualitative literature. Table S9. Location of pain described within the qualitative literature. Table S10. Physiological impacts of pain reported in the qualitative literature. Table S11. Physical functioning impacts reported in the qualitative literature. Table S12. Emotional wellbeing impacts of pain within the qualitative literature. Table S13. Activity of daily living impacts reported in the qualitative literature. Table S14. Negative sleep impacts described in the qualitative literature. Table S15. Social impacts of pain reported in the qualitative literature. Table S16. Work and financial impacts described in the qualitative literature. Table S17. Coping strategies described in the qualitative literature. Table S18. Unmet treatment needs within the qualitative literature. File S1: Redacted targeted literature review protocol.
