# Supplementary material for: Pain Experience in Oncology: A Targeted Literature Review and Development of a Novel Patient-Centric Conceptual Model
Source: Cancers (Basel). 2025 Nov 25;17(23):3760. doi: 10.3390/cancers17233760 (PMC12691514; doi:10.3390/cancers17233760)
Supplement: Supplementary file 1 [file cancers-17-03760-s001.zip › cancers-3961094-File S1.pdf]

Clarivate™

Supplementary Information: Pain Experience in Oncology: A Targeted Literature Review and Development of a Novel Patient-Centric Conceptual Model

Chloe Carmichael [1], Sophie Van Tomme [2], Jordan Miller [1], Danielle Burns [1], Cecile Gousset [3], Helen Kitchen [1], Harriet Makin [1], Natalie V. J. Aldhouse [1] and Paul Cordero [4,*]

[1] Clarivate Analytics, London EC3A 8BE, UK

[2] Sanofi, 1105 BP Amsterdam, The Netherlands

[3] Sanofi, 94255 Gentilly, France

[4] Sanofi, Reading RG6 1PT, UK

* Correspondence: paul.cordero@sanofi.com

Contents of file: Redacted targeted literature review protocol.

Protocol: Targeted qualitative literature review

OBJECTIVE

To conduct a targeted review of published qualitative literature to explore how patients distinguish and describe the types of pain in the context of oncology.

1.1. Data sources

1.1.1. Electronic database searches

The following electronic databases will be searched via Ovid:

- MEDLINE (R) and Epub Ahead of Print, In-Process & Other Non-Indexed Citations and Daily 1946 to present
- Embase[a], 1980 to present
- PsycINFO

Limits will be applied to the database searches to retrieve articles published in the past five years.

Searches will not be limited by country and will explore the patient experience across the globe; differences and similarities across cultures will be noted during analysis. Papers written in non-English languages with an English abstract will be reviewed for eligibility.

A maximum of 30 data sources (journal articles, reports, or transcripts) will be retained for full analysis. Extraction will also include an assessment of study quality using the Critical Appraisal Skills Programme (CASP) tool for qualitative studies(1).

1.1.1.1. Search strings

The database search strings will identify relevant articles (full papers or conference abstracts) indexed on MEDLINE, Embase, and PsycINFO. Three separate search strings will be used to account for differences in syntax within each database. The proposed search strings, including an example search run, are documented in Appendix A and combine terms for cancer AND pain AND qualitative research studies OR conceptual models.

1.1.1.2. Citation screening

An analyst who is trained in literature reviewing will conduct the searches. Results from the database searches will be downloaded into a bespoke database, which will be used to manage citation screening. As an initial step, any duplicated records will be identified and excluded. All remaining citations will then be reviewed by title and, if potentially relevant, abstract (first pass). During first pass, potentially eligible abstracts will be 'tagged' within the database to denote which oncology indications are included.

Upon completion of first pass, seemingly relevant full papers will be sought. Full papers will be examined by an analyst (second pass). Inclusion and exclusion of citations during both passes will be verified by the Project Lead, with any disputes as to eligibility being referred to a third party.

The reasons for exclusion will be documented for each excluded paper, both as a description and using a prospectively designed code system. The reasons for papers being excluded at first and second pass

[a] Source: https://www.embase.com ©2020 Elsevier Life Sciences IP Limited. Embase is a trademark of Elsevier Life Sciences IP Limited, used under license.

(according to the eligibility criteria) will be summarized in a Preferred Reporting Items for Systematic Reviews and Meta-Analyses (PRISMA) flow-chart.

Rules for late papers

There are occasions when publications are delayed or ultimately unavailable. In order to avoid the review being delayed, if papers are not currently available, a study 'end date' will be agreed. All relevant publications received by this date will comprise the dataset for extraction. A tabular record of non-received publications will be maintained.

1.1.2. Hand-searching

Hand-searching is used as a supplementary measure to ensure that all relevant studies are included in the literature review.

1.1.2.1. FDA and EMA websites

Reports from relevant FDA and European Medicines Agency (EMA) meetings/workshop/forums (e.g., 'Voice of the Patient' meetings') will be identified using targeted searches of the FDA (https://www.fda.gov) and EMA websites (https://www.ema.europa.eu/en) for the following terms on each website:

- Cancer AND pain
- [FDA only] Cancer AND "Voice of the patient"

Advanced search limits will be applied to the FDA website when searching for 'Cancer AND pain' to identify the following:

- Center: Center for Drug Evaluation and Research
- Regulated Product: Drugs or Medical Devices.

Similar advanced search limits will also be applied to the EMA website search:

- Include documents: Yes
- Categories: Human
- Topic: Data on Medicines, Regulatory and procedural guidance, Research and development, Scientific advice OR Scientific Guidance

1.1.2.2. Reference lists

Reference lists of included studies will be reviewed for eligible publications.

1.2. Eligibility criteria

Eligibility criteria presented in Table S1 will be applied to all articles/reports identified in the literature search. Any deviations from the eligibility criteria will be documented and rationale will be provided in the final study report.

Table S1. Stage 1 Targeted Qualitative Literature review: Eligibility criteria.

Criteria	Include	Exclude
Population	• Adults diagnosed with any type of cancer	• Studies enrolling pediatric participants only<br>o Studies with mixed populations will be eligible if data are reported for adults and pediatrics separately
Intervention/ Comparator	• No restrictions	• No restrictions.

Criteria	Include	Exclude
Study design	• Studies with qualitative methodology (e.g., Qualitative interviews or focus groups)	• Studies with quantitative methodology only<br>• Case reports<br>• Review papers will not be eligible for inclusion, but highly relevant review papers (i.e., those summarizing qualitative research) will be reviewed to determine whether any of the reviewed primary studies are eligible for inclusion via handsearching methods.
Outcomes	• Patient experience of cancer-associated pain<br>• Patient descriptions of patient's cancer pain and associated health-related quality of life impacts of cancer pain.<br>o Studies reporting clinician or caregiver descriptions of the patient experience of cancer pain will be flagged[b]<br>• Outcomes may be reported as direct quotes or author summaries of collected data	• Patient experience of other symptoms and impacts of cancer or cancer treatments (i.e., not pain)<br>• Quantitative results only (e.g., COA measure scores)<br>o Studies using multiple-choice survey methodology will be flagged for discussion and highly relevant papers may be included if there is a lack of fully qualitative research.
Publication language	• English<br>• Non-English publications with English titles and abstracts	• Non-English publications with Non-English titles/abstracts
Date of publication	• Published in the last 5 years (2018-2023)	• Published prior to the last 5 years (2017 or earlier)

Abbreviations: COA, clinical outcomes assessment.

1.3. Data extraction

Data extraction will be conducted by the research analyst(s) and quality checked by the project lead for completeness; if any discrepancies are noted, the Project Lead will continue to review extracted data until the issues are resolved. For each included study, the following key information will be extracted into a data extraction table (DET):

- Study citation
- Population/sample
  - Cancer type reported (e.g., bladder, breast, colorectal)
  - Disease stage (e.g., Stage 1, 2, 3 or 4)
  - Treatment history (e.g., duration; regimen characteristics)
  - Sample size
  - Sex
  - Age

[b] These studies may be included, in agreement with Sanofi, if fewer than 30 publications reporting patient descriptions are identified.

© Clarivate 2023

- Country
- Study design
  - Study objective(s)
  - Study setting
  - Study method (e.g. semi-structured interview, focus group)
  - Study conclusions

Any included patient quotes or author descriptions/interpretations will undergo secondary analysis using a semantic, qualitative, directed content analysis techniques aided by ATLAS.ti version 9, which facilitates the coding and organization of data. The analysis will take an experiential, realist approach, focusing on participants' individual perspectives and experiences. The following process of qualitative directed content analysis will be completed to explore any open-ended data:

1) **Immersion in the data:** Each publication will be read by a researcher, and overarching analytic ideas and insights identified.
2) **Coding:** Descriptive codes will be assigned to quotes within the publications. The first few publications to be coded will be reviewed by the project team to ensure codes are applied consistently.
   a) For this study, the Project Lead will review the 1st, 3rd and 15th coded paper to confirm that the codes have been applied consistently. Any disputes will be addressed ahead of Step 3.
3) **Iterative review of codes:** The initial fit of the codes to the data will be revisited throughout the coding process. At this stage, codes may be merged together or split into more detailed codes.
4) **Defining and refining concepts and domains:** The research team will meet to discuss their findings and reflect on the codes applied, and may make further revisions to ensure coding is consistent across multiple coders. Codes will then be organized to identify, define and refine concepts and domains relevant to the research question(s).
5) **Reporting:** Final concepts and domains will be reported in a DET within the final study report alongside quotes identified in the literature, where available. Counts will be used to summarize the number of papers where each concept was reported.

1.4. Assessment of study quality

Extraction will also include an assessment of study quality using the CASP checklist (1) for qualitative studies. When data regarding study quality is extracted, reviewers will be prompted to make comments regarding the potential implications for each identified source of potential bias on the study results. There is no cut-off score to determine whether a study is or is not biased, but low quality studies will be flagged for discussion by the study team regarding potential exclusion if deemed likely to contain inaccurate and/or unreliable results.

© Clarivate 2023

2. Stage 2: Identification or development of a conceptual model

OBJECTIVE

To identify (or develop) a conceptual model reporting the patient experience of cancer pain symptoms based on the qualitative literature.

If any existing conceptual model(s) are identified in the literature, the project team will review this against the concepts reported in the qualitative literature for completeness. If no suitable conceptual models are identified during the qualitative literature review, the concepts extracted from the final articles will inform the development of a new conceptual model. The conceptual model will incorporate any differences in the patient experience by age or stage of infection.

The conceptual model will be designed to be representative of the patient experience of cancer-related pain. Conceptual models can also provide a future blueprint for assessing the content validity and conceptual relevance of PROs. An example conceptual model is presented in Figure 1.

All concepts and relationships will be supported by quotes from the qualitative literature and fully referenced. A key may be implemented to denote specific concepts (e.g., those relating to a specific treatment regimen) and/or to denote the source of the information (e.g., those obtained from a direct patient quote or those obtained via an author summary).

Figure S1. Example conceptual model

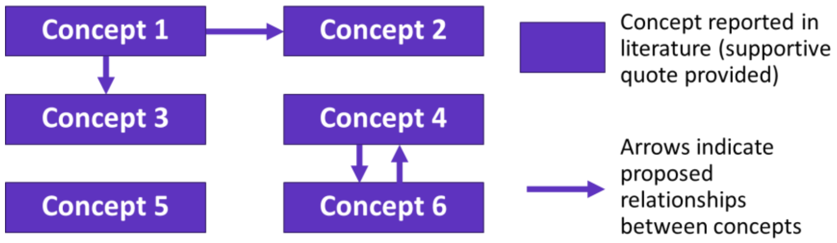

References

1. Critical Appraisal Skills Programme. CASP Qualitative Checklist. Available online: https://casp-uk.net/casp-checklists/CASP-checklist-qualitative-2024.pdf (accessed on 1 June 2023).

Appendix A. Search terms

Medline

Table S2. Ovid MEDLINE(R) and Epub Ahead of Print, In-Process, In-Data-Review & Other Non-Indexed Citations and Daily 1946 to August 02, 2023.

#	Medline terms
1	(Oncol* or cancer* or leuk?emia or tumo* or malignan* or carcinoma* or lymphoma* or myeloma* or sarcoma* or neoplas*).mp.
2	pain.mp.
3	(qualitative research or qualitative study or concept elicitation or qualitative interview* or semi-structured interview* or patient interview* or focus group*).mp.
4	(concept* model* or concept* framework*).mp.
5	3 or 4
6	1 and 2 and 5
7	limit 6 to yr="2018 -Current"

Embase

Table S3. Embase 1980 to 2023 Week 32.

#	Embase terms
1	(Oncol* or cancer* or leuk?emia or tumo* or malignan* or carcinoma* or lymphoma* or myeloma* or sarcoma* or neoplas*).mp.
2	pain.mp.
3	(qualitative research or qualitative study or concept elicitation or qualitative interview* or semi-structured interview* or patient interview* or focus group*).mp.
4	(concept* model* or concept* framework*).mp.
5	3 or 4
6	1 and 2 and 5
7	limit 6 to yr="2018 -Current"

PsycInfo

Table S4. APA PsycInfo 2002 to July Week 3 2023.

#	PsycInfo terms
1	(Oncol* or cancer* or leuk?emia or tumo* or malignan* or carcinoma* or lymphoma* or myeloma* or sarcoma* or neoplas*).mp.
2	pain.mp.
3	(qualitative research or qualitative study or concept elicitation or qualitative interview* or semi-structured interview* or patient interview* or focus group*).mp.
4	(concept* model* or concept* framework*).mp.
5	3 or 4

© Clarivate 2023

#	PsycInfo terms
6	1 and 2 and 5
7	limit 6 to yr="2018 -Current"

© Clarivate 2023
