# Supplementary material for: Pain Experience in Oncology: A Targeted Literature Review and Development of a Novel Patient-Centric Conceptual Model
Source: Cancers (Basel). 2025 Nov 25;17(23):3760. doi: 10.3390/cancers17233760 (PMC12691514; doi:10.3390/cancers17233760)
Supplement: Supplementary file 1 [file cancers-17-03760-s001.zip › cancers-3961094-Supplementary materials.pdf]

Supplementary Information: Pain Experience in Oncology: A Targeted Literature Review and Development of a Novel Patient-Centric Conceptual Model

Chloe Carmichael [1], Sophie Van Tomme [2], Jordan Miller [1], Danielle Burns [1], Cecile Gousset [3], Helen Kitchen [1], Harriet Makin [1], Natalie V. J. Aldhouse [1] and Paul Cordero [4,*]

1 Clarivate Analytics, London EC3A 8BE, UK
2 Sanofi, 1105 BP Amsterdam, The Netherlands
3 Sanofi, 94255 Gentilly, France
4 Sanofi, Reading RG6 1PT, UK
* Correspondence: paul.cordero@sanofi.com

Contents of file: Supplementary tables providing an overview of the quality assessment and methodology of the included studies, the list of eligible studies that were not retained for data extraction and data on pain triggers, descriptors, impacts, coping strategies, and unmet needs, as reported in the literature through direct patient reports and author summaries.

Quality Assessment

Table S1 provides an overview of the CASP quality appraisal conducted for the included studies. As part of the CASP appraisal, the reviewer is asked to record a "yes" (✓), "no" (x) or "can't tell" (?) to each prompt. The checklist was designed to be used as an educational pedagogic tool and therefore numerical values were not assigned to the studies. In addition, studies were not excluded based on the critical appraisal results.

Table S1. Stage 1: CASP quality assessment of included studies

**Publication**	Was there a clear statement of the aims of the research?	Is a qualitative methodology appropriate?	Was the research design appropriate to address the aims of the research?	Was the recruitment strategy appropriate to the aims of the research?	Was the data collected in a way that addressed the research issue?	Has the relationship between researcher and participants been adequately considered?	Have ethical issues been taken into consideration?	Was the data analysis sufficiently rigorous?	Is there a clear statement of findings?	How valuable is the research?
**Adam, 2018 [1]**	✓	✓	✓	✓	✓	✓	✓	✓	✓	✓
**Allsop, 2019 [2]**	✓	✓	✓	✓	✓	✓	✓	✓	✓	✓
**Appleyard, 2018 [3]**	✓	✓	✓	✓	✓	✓	✓	✓	✓	✓
**Barrett, 2023 [4]**	✓	✓	✓	✓	✓	?	✓	✓	✓	✓
**Benali, 2022 [5]**	✓	✓	✓	✓	✓	✓	✓	?	✓	✓
**Cella, 2023 [6]**	✓	✓	✓	✓	✓	?	✓	?	✓	✓
**Ekstedt, 2019 [7]**	✓	✓	✓	✓	✓	✓	✓	✓	✓	✓
**Englid, 2023 [8]**	✓	✓	✓	✓	✓	✓	✓	✓	✓	✓
**Erol, 2018 [9]**	✓	✓	✓	✓	✓	✓	✓	✓	✓	✓
**Everaars, 2021 [10]**	✓	✓	✓	✓	✓	✓	✓	✓	✓	✓

Publication	Was there a clear statement of the aims of the research?	Is a qualitative methodology appropriate?	Was the research design appropriate to address the aims of the research?	Was the recruitment strategy appropriate to the aims of the research?	Was the data collected in a way that addressed the research issue?	Has the relationship between researcher and participants been adequately considered?	Have ethical issues been taken into consideration?	Was the data analysis sufficiently rigorous?	Is there a clear statement of findings?	How valuable is the research?
**Hassankhani, 2023 [11]**	✓	✓	✓	✓	✓	✓	✓	✓	✓	✓
**Hodge, 2022 [12]**	✓	✓	✓	✓	✓	✓	✓	✓	✓	✓
**Koulouris, 2021 [13]**	✓	✓	✓	✓	✓	✓	✓	✓	✓	✓
**Liu, 2018 [14]**	✓	✓	✓	✓	✓	✓	✓	✓	✓	✓
**Maly, 2018 [15]**	✓	✓	✓	✓	✓	✓	✓	✓	✓	✓
**Martin, 2022 [16]**	✓	✓	✓	?	✓	?	✓	?	✓	✓
**Nabulsi, 2023 [17]**	✓	✓	✓	✓	✓	✓	✓	✓	✓	✓
**O'Regan, 2023 [18]**	✓	✓	✓	✓	✓	✓	✓	✓	✓	✓
**Restivo, 2023 [19]**	✓	✓	✓	✓	✓	✓	✓	✓	✓	✓
**Schumacher, 2021 [20]**	✓	✓	✓	✓	✓	✓	✓	✓	✓	✓
**Smith, 2023 [21]**	✓	✓	✓	✓	✓	✓	✓	✓	✓	✓
**Vestergaard, 2023 [22]**	✓	✓	✓	✓	✓	✓	✓	✓	✓	✓

Publication	Was there a clear statement of the aims of the research?	Is a qualitative methodology appropriate?	Was the research design appropriate to address the aims of the research?	Was the recruitment strategy appropriate to the aims of the research?	Was the data collected in a way that addressed the research issue?	Has the relationship between researcher and participants been adequately considered?	Have ethical issues been taken into consideration?	Was the data analysis sufficiently rigorous?	Is there a clear statement of findings?	How valuable is the research?
**Walsh, 2022 [23]**	✓	✓	✓	✓	✓	✓	✓	✓	✓	✓
**Wei, 2022 [24]**	✓	✓	✓	✓	✓	?	✓	✓	✓	✓
**Whisenant, 2021 [25]**	✓	✓	✓	✓	✓	✓	✓	✓	✓	✓
**Xu, 2019 [26]**	✓	✓	✓	✓	✓	✓	✓	✓	✓	✓
**Yeager, 2018 [27]**	✓	✓	✓	✓	✓	✓	✓	✓	✓	✓
**Yeager, 2023 [28]**	✓	✓	✓	✓	✓	✓	✓	✓	✓	✓

Methodology of included studies

Table S2 provides an overview of the methodology and analytic approaches applied by the 28 included studies.

Table S2. Methodology of the included studies.

Study	Study objective(s)	Methods	Analytic approach
**Adam, 2018 [1]**	To explore current pain management strategies used by patients, caregivers and professionals and to investigate opportunities for digital technologies to enhance cancer pain management.	Qualitative semi-structured interviews (face-to-face or telephone)	Thematic analysis
**Allsop, 2019 [2]**	To describe palliative care patients' perspectives on managing and talking about pain and the role of technology in their lives.	Qualitative semi-structured interviews (face-to-face)	Framework analysis
**Appleyard, 2018 [3]**	To assess how older people self-manage their cancer pain at home and what their experiences are in relation to potential facilitators, personal strengths, and coping strategies.	Qualitative semi-structured interviews (face-to-face)	Interpretative phenomenological analysis
**Barrett, 2023 [4]**	To develop new PRO scales, using an existing item bank, to measure the patient experience of pain and fatigue in soft tissue sarcoma (STS).	Qualitative semi-structured interviews	Thematic analysis
**Benali, 2022 [5]**	To explore the expectations and experiences of women undergoing internal radiation for cervical cancer at the brachytherapy unit of the Radiation Oncology Department of the National Institute of Oncology in Rabat, Morocco.	Qualitative semi-structured interviews (face-to-face)	Thematic analysis
**Cella, 2023 [6]**	To evaluate the experience of patients with acute myeloid leukemia (AML) in	Qualitative semi-structured interviews (telephone)	NR

	remission following hematopoietic stem cell transplant (HSCT)		
**Ekstedt, 2019 [7]**	To evaluate possible critical components, facilitators, and hindrances for improved knowledge about pain management, in not hospitalized adult oncology patients with pain from bone metastasis participating in a pain management intervention.	Embedded qualitative interviews in an RCT of a psychoeducational pain management intervention.	Content analysis
**Englid, 2023 [8]**	To explore and describe patients' experiences of pain treatment in the perioperative period after surgery for pancreas cancer.	Qualitative semi-structured interviews (face-to-face)	Content analysis
**Erol, 2018 [9]**	To explore the pain experiences of patients with advanced cancer and how they manage with pain, and to present a view of pain management approaches of nurses from the perspectives of the patients.	Qualitative semi-structured interviews (face-to-face)	Colaizzi's phenomenological method
**Everaars, 2021 [10]**	To explore women's experiences after breast surgery with scar characteristics and symptoms, and its impact on their health-related quality of life (HRQoL).	Qualitative semi-structured interviews (face-to-face)	Directed content analysis
**Hassankhani, 2023 [11]**	To comprehend the effect of social–cultural context on the pain experiences of Iranian cancer patients and to cover any potential gap.	Qualitative semi-structured interviews (face-to-face)	Content analysis
**Hodge, 2022 [12]**	To explore the cultural constructs of pain, a series of focus groups were held among adult American Indian (AI) cancer survivors and their	Focus groups	NR

	caregivers in the Southwest USA.		
**Koulouris, 2021 [13]**	To assess the burden of pain in patients and caregivers of patients with pancreatic cancer. Secondly, to assess whether endoscopic analgesia is acceptable during the terminal illness.	Qualitative semi-structured interviews (face-to-face or online)	Thematic analysis
**Liu, 2018 [14]**	To examine the perception of cancer patients with breakthrough pain in the Northwest of China.	Qualitative semi-structured interviews (face-to-face)	Inductive thematic analysis
**Maly, 2018 [15]**	To add to the understanding of cancer pain and perceived control over pain in African Americans, from the patients' perspective.	Qualitative semi-structured interviews (face-to-face)	Phenomenological approach with line-by-line analysis
**Martin, 2022 [16]**	To characterize pain and its impact in patients with locally advanced (la) or metastatic urothelial carcinoma (mUC) treated in the first-line setting, and to assess the content validity of the Brief Pain Inventory – Short Form (BPI-SF) worst pain item in this population.	Qualitative semi-structured interviews (telephone)	NR; references Braun and Clarke's thematic analysis
**Nabulsi, 2023 [17]**	To understand patient attitudes towards opioids for hematologic malignancies (HM) pain management, particularly among historically marginalized populations	Qualitative semi-structured interviews (face-to-face)	Framework analysis
**O'Regan, 2023 [18]**	To better understand cancer survivors' pain management experiences after curative-intent treatment.	Qualitative semi-structured interviews (online or telephone)	Thematic analysis
**Restivo, 2023 [19]**	To explore cancer patients' experience of pain and	Qualitative semi-structured interviews (face-to-face)	Grounded Theory

	coping strategies as well as beliefs and representations associated with pain, pain management and treatments.		
**Schumacher, 2021 [20]**	To examine patients' pain experiences as they occurred in real time during the study pain management intervention.	Embedded qualitative interviews within a randomized controlled trial of a psychoeducational pain management intervention.	Interpretative emergent approach
**Smith, 2023 [21]**	To explore experiences of women with persistent pain following breast cancer treatment, including their perceptions about the cause of their pain, how they manage their pain and their interactions with healthcare providers related to their pain during and following breast cancer treatment.	Qualitative semi-structured interviews or focus groups (face-to-face or telephone)	Framework analysis
**Vestergaard, 2023 [22]**	To examine the patient's perspective on calcium electroporation (CaEP) treatment of skin tumors in patients with a variety of primary cancer diagnoses, as well as the treatment's impact on HRQoL.	Qualitative semi-structured interviews (face-to-face or telephone)	Thematic analysis
**Walsh, 2022 [23]**	To understand nuances of the pain experience, if experiences vary by patient characteristics, and to guide the development of adjuvant endocrine therapy (AET) pain specific resources and support for patients with early-stage breast cancer	Qualitative semi-structured interviews (face-to-face or telephone)	Thematic content analysis
**Wei, 2022 [24]**	To investigate patients' experiences after lung cancer surgery and analyze whether the perception of postoperative symptoms among the healthcare	Qualitative semi-structured interviews (face-to-face)	Grounded theory

	providers differed from that reported by patients.		
**Whisenant, 2021 [25]**	To identify symptoms experienced and to determine the content domain for a PRO measuring symptom burden for patients who had received standard of care Chimeric antigen receptor (CAR) T-cell therapy for advanced B-cell lymphoid malignancies.	Qualitative semi-structured interviews (face-to-face)	Content analysis
**Xu, 2019 [26]**	To describe the experiences of pain acceptance in Chinese cancer patients with chronic pain.	Qualitative semi-structured interviews (face-to-face)	Colaizzi's seven-step method
**Yeager, 2018 [27]**	To explore the symptom experience of blacks with advanced cancer by describing the symptoms these individuals experience and how these symptoms affect their day-to-day lives.	Qualitative semi-structured interviews (face-to-face)	Content analysis
**Yeager, 2023 [28]**	To describe the day-to-day experience of living with pain and the experiences of taking opioids for pain management among Black individuals with cancer prescribed with long-acting opioids.	Qualitative semi-structured interviews (face-to-face)	Content analysis

Eligible publications not retained for data extraction

Table S3 presents details of the eligible publications that were not retained for data extraction during screening.

Table S3. Eligible publications that were not retained for data extraction.

First author, year	Title
**Al-Ghabeesh, 2020**	Barriers to effective pain management in cancer patients from the perspective of patients and family caregivers: A qualitative study
**Alsaraireh, 2022**	Cancer pain management program: Patients' experiences-A qualitative study
**Andersson, 2022**	Perceptions of Experiences of Recovery After Pancreaticoduodenectomy - A Phenomenographic Interview Study
**Azizoddin, 2021a**	Cancer pain self-management in the context of a national opioid epidemic: Experiences of patients with advanced cancer using opioids
**Azizoddin, 2021b**	Leveraging mobile health technology and research methodology to optimize patient education and self-management support for advanced cancer pain
**Bharmal, 2018**	How to address the challenges of evaluating treatment benefits-risks in rare diseases? A convergent mixed methods approach applied within a Merkel cell carcinoma phase 2 clinical trial
**Birgin, 2023**	Development of a conceptual framework to detect perioperative symptom burden following abdominal surgery for cancer
**Brooks, 2020**	Palliative Care's Role Managing Cancer Pain During the Opioid Crisis: A Qualitative Study of Patients, Caregivers, and Clinicians
**Brown, 2020**	Health-Related Quality of Life After Lobectomy for Lung Cancer: Conceptual Framework and Measurement
**Catt, 2019**	Patients' and partners' views of care and treatment provided for metastatic castrate-resistant prostate cancer in the UK
**Claydon, 2023**	Patient experiences of left-sided colorectal resection by robotic, conventional laparoscopic and open approaches: a qualitative study
**Cohen, 2023**	Patient Perceptions Regarding Ciltacabtagene Autoleucel Treatment: Qualitative Evidence From Interviews With Patients With Relapsed/Refractory Multiple Myeloma in the CARTITUDE-1 Study
**Cuesta-Briand, 2021**	Delays in the Pathway to Cancer Diagnosis in Samoa: A Qualitative Study of Patients' Experiences
**Curry, 2019**	Percutaneous venting gastrostomy/gastrojejunostomy for malignant bowel obstruction: a qualitative study
**Cusimano, 2020**	Supported self-management as a model for end-of-life care in the setting of malignant bowel obstructions: a qualitative study

**Cyr, 2023**	Gynecological cancer survivors' experiences of dyspareunia and factors influencing care-seeking behavior: A qualitative study
**De, 2023**	Feasibility and pilot testing of a personalized eHealth intervention for pain science education and self-management for breast cancer survivors with persistent pain: a mixed-method study
**Demirci, 2022**	Effect of foot massage on upper extremity pain level and quality of life in women who had a mastectomy operation: A mixed-method study
**Edwards, 2018**	How do patients with cancer pain view community pharmacy services? An interview study
**Ehrlich, 2019**	Cancer Pain Social Processes and Pain Management in Home Hospice Care
**Eisen, 2021**	The Needs and Experiences of Patients on Pain Education and the Clinical Reasoning of Physical Therapists Regarding Cancer-Related Pain. A Qualitative Study
**Farrell, 2018**	Understanding the impact of chemotherapy on dignity for older people and their partners
**Giannitrapani, 2021**	How Patients and Providers Weigh the Risks and Benefits of Long-Term Opioid Therapy for Cancer Pain
**Glynn, 2020**	Exploring Cancer Patients' Experiences of an Online Mindfulness-Based Program: A Qualitative Investigation
**Graczyk, 2018**	Why patients are afraid of opioid analgesics: A study on opioid perception in patients with chronic pain
**Grocott, 2023**	Breast cancer patient experiences of perioperative distress and anxiety: A qualitative study
**Hinata, 2023**	Encouraging outpatients in an acute hospital for the relief of cancer-related pain: a qualitative study
**Ho, 2020**	Morphine use for cancer pain: A strong analgesic used only at the end of life? A qualitative study on attitudes and perceptions of morphine in patients with advanced cancer and their caregivers
**Hodge, 2020**	We Don't Talk about It: Cancer Pain and American Indian Survivors
**Hollander-Mieritz, 2019**	Comparing the patients' subjective experiences of acute side effects during radiotherapy for head and neck cancer with four different patient-reported outcomes questionnaires
**Humphrey, 2023**	Development of recommendations to improve patient experiences of brachytherapy for locally advanced cervical cancer

**Kelleher, 2022**	Hybrid-delivered cognitive behavioral symptom management and activity coaching intervention for patients undergoing hematopoietic stem cell transplant: Findings from intervention development and a pilot randomized trial
**Koulouris, 2021**	Patients', carers', and clinicians' beliefs and ideas towards endoscopic analgesia for unresectable pancreatic cancer: a qualitative study using semi-structured interviews
**Li, 2020**	Patient perspectives on frame versus mask immobilization for gamma knife stereotactic radiosurgery
**Lovric, 2018**	A Qualitative Study Exploring Patients' Expectations and Experiences of the Localization Event as Part of Radiation Therapy
**Luckett, 2022**	Patient and carer experiences of pain care in an Australian regional comprehensive cancer care setting: a qualitative study
**Maglia, 2022**	The Network That Unites a Qualitative Study on Clinical Psychological Intervention for Women with a History of Breast Cancer and Chronic Pain
**Marshall, 2022**	Barriers to Adequate Pain Control and Opioid Use Among Cancer Survivors: Implications for Nursing Practice
**Meghani, 2020**	When Patients Take Charge of Opioids: Self-Management Concerns and Practices Among Cancer Outpatients in the Context of Opioid Crisis
**Moris, 2022**	Quality of Life Associated with Open vs Minimally Invasive Pancreaticoduodenectomy: A Prospective Pilot Study
**Nedjat-Haiem, 2020**	Moving closer to death: understanding psychosocial distress among older veterans with advanced cancers
**Ochoa, 2019**	Finding myself as a cervical cancer survivor: A qualitative study
**O'Connor, 2021**	Pain assessment and registration in medical oncology clinics: operationalised through the lens of health care professionals and patients
**Orujlu, 2022**	Barriers to cancer pain management from the perspective of patients: A qualitative study
**Pham, 2021**	"I Wish I Could Die So I Would Not Be in Pain": A Qualitative Study of Palliative Care Needs Among People With Cancer or HIV/AIDS in Vietnam and Their Caregivers
**Prabandari, 2022**	"Alas ... my sickness becomes my family's burden": A nested qualitative study on the experience of advanced breast cancer patients across the disease trajectory in Indonesia
**Rafii, 2021**	Barriers to Effective Cancer Pain Management in Home Setting: A Qualitative Study
**Renman, 2022**	Attitudes to and Experiences of Physical Activity After Colon Cancer Diagnosis Amongst Physically Active Individuals - A Qualitative Study

**Rini, 2018**	A qualitative study of patient and provider perspectives on using web-based pain coping skills training to treat persistent cancer pain
**Sales-Wuillemin, 2020**	Pain and suffering: Cross-representations between oncologists and patients, a qualitative study
**Samuel, 2018**	Racial differences in symptom management experiences during breast cancer treatment
**Shammas, 2022**	Dissatisfaction After Post-Mastectomy Breast Reconstruction: A Mixed-Methods Study
**Sheill, 2018**	The views of patients with metastatic prostate cancer towards physical activity: a qualitative exploration
**Singh, 2018**	Experiences of urban African Americans with cancer pain
**Shi, 2021**	Quantitative-qualitative analyses of patient-reported pain response after palliative radiation therapy
**Somers, 2018**	An mHealth Pain Coping Skills Training Intervention for Hematopoietic Stem Cell Transplantation Patients: Development and Pilot Randomized Controlled Trial
**Thomas, 2020**	Exploring the impact of lymphoedema on individuals and if lymphatic venous anastomosis surgery effects perceptions on quality of life: A qualitative study
**Tola, 2022**	Perception of music and its cultural elements on acute post-mastectomy pain management among Nigerian women: an exploratory qualitative study
**Tsui, 2022**	Experiences of Losing Bowel Control After Lower Anterior Resection With Sphincter Saving Surgery for Rectal Cancer: A Qualitative Study
**Van Cleave, 2019**	The development, usability, and reliability of the Electronic Patient Visit Assessment (ePVA) for head and neck cancer.
**Van Haren, 2021**	Qualitative Evaluation of the Influence of Acute Oxaliplatin-Induced Peripheral Neuropathy on Quality of Life and Activities of Daily Life
**Walker, 2022**	Use of complementary and integrative health in cancer pain management among patients undergoing cancer treatments: a qualitative descriptive study
**Watson, 2020**	Qualitative Findings on the Impact of Disease in Epstein-Barr Virus-Driven Post-Transplant Lymphoproliferative Disease Patients, as Measured by EQ-5D, SF-36, and the FACT-LYM
**Williams, 2020**	In Search of the Optimal Outcome Measure for Patients with Advanced Cancer and Gastrointestinal Obstruction: A Qualitative Research Study
**Winger, 2020**	Enhancing meaning in the face of advanced cancer and pain: Qualitative evaluation of a meaning-centered psychosocial pain management intervention

**Wright, 2019**	Patient Patterns and Perspectives on Using Opioid Regimens for Chronic Cancer Pain
**Xu, 2023**	Cultural factors affecting Chinese migrants' perceptions and responses to cancer pain and its pharmacological management: A convergent mixed-method study
**Yeon, 2022**	Tearing down the barriers to exercise after mastectomy: a qualitative inquiry to facilitate exercise among breast cancer survivors

Pain Triggers

Table S4 provides an overview of the symptom triggers reported in the literature through direct patient reports and author summaries.

Table S4. Pain triggers reported in the qualitative literature.

Trigger	Number of sources	Sources	Example supporting quotes
Procedure	12	[4, 5, 8, 10-13, 20-22, 24, 26]	– *"I had nine bone marrow biopsies. It was extremely painful. Every time I underwent biopsy, I die again and again." [11]* [Not reported]<br>– Author commentary: Pain was a major issue for most once the anesthesia wore off, and participants reported unrelieved pain lasting for hours and even days after the procedure. *[5]* [Cervical cancer]<br>– *"In the beginning I had a lot of trouble. Yes! Maybe even more […] the pain was more extreme, yes worse, certainly just after surgery." [10]* [Breast cancer]<br>– Author commentary: [describing the experience of high dose-rate Brachytherapy] Participants were overwhelmed by the "hell" they experienced when the applicators were inserted. *[5]* [Cervical cancer]
Treatment	9	[4, 6, 16, 19-21, 23, 26, 27]	– *"I could tolerate the pain that surgery and chemotherapy induced at first. But now it's too painful to endure … It feels like pins and needles. I don't have a way to feel better." [26]* [Lung cancer]<br>– *"It's miserable. Being in pain is just miserable, and that's what tamoxifen makes you." [23]* [Breast cancer]<br>– Author commentary: Three women spoke of the pain from skin burns that they received from radiation treatment for their cancers (lung, breast, or cervical), and each speculated that they had received too much radiation. Ms. C said the burn felt like having hot grease thrown on her skin. *[27]* [Not reported]
Tumor or metastases	7	[4, 9, 11, 16, 20, 22, 27]	– *"I have a (tumor) on my shoulder blade here at the back, so it's a bit difficult to lie down properly without it hurting. It also feels very strange—it's like a foreign body—It just has to go." [22]* [Not reported]<br>– Author commentary: Nancy had pain in one arm throughout the study, associated with a tumor in her brachial plexus and lymphedema. *[20]* [Not reported]<br>– *"I felt mad when I had pain. My pain is because of a mass in my abdomen. My abdomen is enlarged and I feel myself uncomfortably full." [9]* [Not reported]
Movement	6	[3, 8, 10, 19, 24, 26]	– *"I can't bear any stimulation, or any small activity, thought, discussion, or movement that will trigger pain or make it worse." [26]* [Lung cancer]

Trigger	Number of sources	Sources	Example supporting quotes
			– [describing the experience of lung cancer surgery] *"Maybe I cannot bear the pain; however, sometimes the pain is significantly severe. I experienced severe pain with the chest drainage tube. It was significantly painful to move around." [24]* [Lung cancer]<br>– *"When I make certain movements, it pulls. The fact that I'm limited in my actions, which I did not have before... for example when I went for a swim... and that's actually also the case with skiing […]" [10]* [Breast cancer]
Time of day	5	[6, 11, 19, 24, 27]	– *"No, it was more like at the end of the day when I'm finally relaxing that I would feel the pain in my legs." [6]* [Acute myeloid leukemia]<br>– Author commentary: Others described trouble sleeping because their cough was worse at night than during the day and that the cough aggravated their pain. Individuals with lung cancer and breast cancer who had metastatic disease in their lungs were most likely to recount the co-occurring symptoms of pain, coughing, and poor sleep. *[27]* [Not reported]
Negative thoughts	3	[11, 20, 26]	– *"When I'm worried or sad, I'm in a lot of pain. When I get bad news, I feel like I'm going crazy." [11]* [Not reported]
Passing urine or stool	3	[16, 20, 28]	– *"I was having pain upon urination and just strange cramping sensations, and just some other things that I couldn't quite explain that was not going away. It was almost like it would come and go. It would be like…it would be like an achy sensation just like really sore, achy and I couldn't explain it." [16]* [Not reported]
Walking	3	[1, 3, 14]	– *"'I could walk when I went to Beijing three months ago but I cannot walk now because this will evoke pain." [14]* [Not reported]
Eating	2	[13, 14]	– *"As soon as I take anything inside me like this morning after breakfast I felt quite uncomfortable and bloated but then I haven't been eating a great deal so that was just too much if you like in in one day, I don't know. My porridge this morning was really quite uncomfortable to, to live with for a little while...I'm also scared of eating because of what it does to me." [13]* [Pancreatic cancer]
Physical activity or overexertio n	2	[1, 20]	– *"Up until two years ago, I led a very active life, (…) I find, as long as I'm doing nothing, the hundred a day [of long-acting morphine] is fine, but I can't sit about doing nothing. So I try to do as much as I can, but an hour, an hour and a half and I have to take morphine, (…) then I have to relax until the morphine kicks in and I'm not feeling any pain anymore." [1]* [Prostate cancer]
Weather	2	[6, 10]	– *"If it's colder [...] then it really affects my scar, a little like muscle pain plus." [10]* [Breast cancer]

Trigger	Number of sources	Sources	Example supporting quotes
Sneezing or coughing	2	[6, 27]	– Author commentary: Others described trouble sleeping because their cough was worse at night than during the day and that the cough aggravated their pain. Individuals with lung cancer and breast cancer who had metastatic disease in their lungs were most likely to recount the co-occurring symptoms of pain, coughing, and poor sleep. *[27]* [Not reported]
Inactivity or sedentary behavior	2	[20, 23]	– *"I can kind of tolerate the bone pain because it does-- it is when I wake up or sit for a period of time and then it does get better as I move." [23]* [Breast cancer]

Pain Descriptors

Overview

Table S5 provides an overview of the descriptors for pain across each of the treatment stages identified within the qualitative literature.

Table S5. Pain descriptors across treatment stages.

Pain descriptor	Pain experienced pre-treatment/ diagnosis (n=5)*	Pain experienced while living with cancer/during active treatment (n=23)*	Pain experienced post-treatment (n=6)*	Continuing pain in cancer survivors (n=2)*
Sharp/stabbing/ shooting	✓ (n=2)	✓ (n=8)	✓ (n=1)	✓ (n=1)
Pounding/hammering/ throbbing		✓ (n=2)		✓ (n=1)
Cramping or spasm		✓ (n=4)	✓ (n=2)	
Deep or aching		✓ (n=5)	✓ (n=2)	
Neuropathic (e.g., numbness, tingling)		✓ (n=5)		
Bone pain		✓ (n=6)		
Breakthrough pain		✓ (n=2)		
Burning/searing		✓ (n=4)		
Crushing/gripping		✓ (n=4)		
Dull		✓ (n=1)		

Pain descriptor	Pain experienced pre-treatment/ diagnosis (n=5)*	Pain experienced while living with cancer/during active treatment (n=23)*	Pain experienced post-treatment (n=6)*	Continuing pain in cancer survivors (n=2)*
Non-specific discomfort		✓ (n=5)		
Punishing or sickening		✓ (n=1)		
Tender to touch/inflamed/warm		✓ (n=2)		
Tightness/tugging/ tearing			✓ (n=2)	
"Worse than childbirth"		✓ (n=1)		

*Counts are not mutually exclusive.

Frequency

Table S6 provides an overview of the frequency of pain, as described by patients and authors in the qualitative literature.

Table S6. Frequency of pain experienced in the qualitative literature.

Frequency	Number of sources	Sources	Example supporting quotes
Daily	5	[10, 16, 18, 23, 28]	– *"Every day I'm battling pain which has totally changed me. I loved the way I was raised, I loved the priorities, I loved working hard, I loved physical activity, I loved being out in the woods, I like hiking, I like going camping. And all of those things are being taken from me." [18]* [Breast cancer]<br>– *"My pain is — is constant. I mean, every day.…And so psychologically, I had to go to God to be able to manage this. Sometimes, it's unbearable." [28]* [Not reported]
Intermittent	4	[13, 14, 16, 23]	– *''I cannot sleep well at night, I have an intermittent pain here (pointed to lower abdomen) and I can only sleep 3-4 hours. The pain wakes me up." [14]* [Not reported]
Persistent	3	[11, 16, 20]	– *"There's achiness, there's pain…. It's consistent, consistent pain." [16]* [Bladder cancer]
Unpredictable	2	[1, 14]	– *''Pain is a life-threatening symptom for me. It makes my life miserable, I no longer can do things like a normal person [sighed]. I can't even take a walk outside the ward because I don't know when the pain will attack' '[14]* [Not reported]

Duration

Table S7 summarizes the duration of pain, as described by patients and authors in the qualitative literature.

Table S7. Duration of pain experienced in the qualitative literature.

Duration	Number of sources	Sources	Example supporting quotes
Chronic	10	[2, 6, 11, 12, 15, 18, 20, 21, 26, 28]	– *"I mean some days I could manage the pain quite well and other days I couldn't manage it at all, I felt I didn't have much control over it; it was just fluctuating throughout the course of the day, and some days it just stayed steady and other days it just, but as I say I was always I'm always in pain, it's always there." [2]* [Head and neck cancer]<br>– Author commentary: According to participants, cancer pain is persistent pain that never subsides or improves over the course of the illness. *[11]* [Not reported]<br>– Author commentary: Paul had chemotherapy-related neuropathic pain in his feet and legs that remained essentially unchanged, despite multiple management strategies. *[20]* [Not reported]<br>– *"My pain is — is constant. I mean, every day….And so psychologically, I had to go to God to be able to manage this. Sometimes, it's unbearable." [28]* [Not reported]
Days	2	[5, 6]	– *"I primarily have two to three days of headache after the maintenance therapy. So it's only one to two days, and you really can live with that. That's not so bad." [6]* [Acute myeloid leukemia]
Short-lived	2	[7, 13]	– *"And, um, trying to handle a pain like that…it's pretty intense when it comes along, but then it's gone again in a tenth of a second. I learned to sort of just sit still for the short time it lasted…move with caution." [7]* [Not reported]
Variable	2	[20, 23]	– Author commentary: They could be associated with any of the sources of pain described above and could last for varying lengths of time, ranging from a few minutes or hours to several days or weeks. *[20]* [Not reported]
Weeks	2	[6, 22]	– *"It got better around the time when the lab results got more stable. So, after fourteen days my joint and bone pain was getting better and eventually, they disappeared altogether." [6]* [Acute myeloid leukemia]

Severity

Table S8 provides an overview of the descriptions of the severity of pain, as described by patients and authors in the qualitative literature.

Table S8. Severity of pain experienced in the qualitative literature.

Severity	Number of sources	Sources	Example supporting quotes
Severe	24	[1-3, 5-11, 13-18, 20, 21, 23-28]	– *"I can get these pulse pains as well where they're really, it feels like something has just grabbed my liver and squeezed it hard, squeezing, and it's really, really chronically painful, and then it'll just suddenly stop, and it leaves me feeling bruised, quite warm, quite wet around that area, so it's like it's bled out but it's not bleeding, it just goes off after a while." [2]* [*Head and neck cancer]*<br>– *"It was very difficult and painful. It felt like a punishment." [5] [Cervical cancer]*<br>– *"The pain score can be up to 8, I really suffer from that.'' [14] [Not reported]*<br>– *"But several times when I was suffering from severe pain, I sweated all over my body and the pain knocked me out then I fell asleep.'' [14] [Not reported]*<br>– *"It seems like the pain has gotten really bad since I've started the chemo, whereas before it was a marginal or minimal pain that way." [16] [Bladder cancer]*
Unbearable	16	[2, 3, 5, 8, 9, 11, 14-17, 20, 21, 24, 26-28]	– *"My pain is becoming unbearable. When I feel such intense pain, it is like I'm dying of pain. I feel like I'm about to die." [11]* [Not reported]<br>– *''It's horrible. This unbearable pain experience makes me can do nothing but cry, and I want to die rather than continue suffering from this pain. I cannot bear it.'' [14]* [Not reported]<br>– *"I felt like I didn't even want to live, the pain was so bad.'' ''I was in so much pain. I couldn't bear it anymore that's why I had to go to the hospital. If I could let somebody feel the pain, they would say how did you deal with this for so long and the only thing I could say is by the grace of God. I really wanted to die. I really meant that. I really wanted to die.'' [15]* [Not reported]<br>– *"I feel like it's better to receive euthanasia when pain is extremely intense. It's meaningless to live for extra years. I prefer to die rather than live with endless pain." [26]* [Lung cancer]<br>– *"But it can get to a point that it shatters your mind. It was like your mind just shot. The pain can be so excruciating. And I said to myself… I can understand why some people just give up and die instead of go through it." [27]* [Not reported]
Variable	7	[1, 2, 6, 8, 12, 16, 20]	– *"I mean some days I could manage the pain quite well and other days I couldn't manage it at all, I felt I didn't have much control over it; it was just fluctuating throughout the course of the day, and some days it just stayed steady and other days it just, but as I say I was always I'm always in pain, it's always there". [2]* [Head and neck cancer]<br>– *"Ronald described the cycle of treatment-related pain as a "roller coaster" and explained: "As I go through this chemo treatment, the first week isn't too bad; the second week is a little worse; the third*

Severity	Number of sources	Sources	Example supporting quotes
			week I feel almost perfect, just great… and then I take the chemo again, and it starts over." [20] [Not reported]<br>– *"It varies. The further I get away from my bone marrow transplant, the less [pain] I have. Right now, it might be three out of seven days. Before, it was every day. every day I woke up and it was there. Now, it's going away." [6]* [Acute myeloid leukemia]
Mild	6	[6, 9, 19, 21, 23, 24]	– Author commentary*:* while others reported experiencing mild pain, e.g., a headache or back pain. *[9]* [Not reported]<br>– *"When I woke up after the operation, I felt a little pain, mainly chest wound pain, but the pain was not significantly severe. When the chest drainage tube was removed, I could move and walk more freely; however, there was no significant difference compared to the pain experienced previously. The pain usually started at 6 p.m. and was more evident at night." [24]* [Lung cancer]<br>– *"Kind of light headaches every once in a while, intermittently. That was pretty much it." [6]* [Acute myeloid leukemia]

Location

Table S9 provides an overview of the location of pain experienced by patients in the qualitative literature. Most data were derived from single indication publications [4-6, 8, 10, 13, 16, 21, 23-25] or from studies with a mixed population where the indication was specified [1, 7, 22, 26, 27].

Table S9. Location of pain described within the qualitative literature.

Location	Number of sources	Sources	Example supporting quotes
Back	11	[5, 6, 9, 11, 16, 19, 20, 22, 27]	– *"My back felt so uncomfortable, like it was broken." [5]* [Cervical cancer]<br>– *"Now my pain is terrible and affects my back and neck. This bothers me considerably. The pain begins in the middle of my back and spreads to the shoulder, and then to the fingertips." [11]* [Not reported]<br>– *"I can't even touch my back. It's still burning. It was as if I had been electrocuted." [11] [Not reported]*<br>– *"And then I just started having really bad back pain out of nowhere that started getting worse". [16]* [Bladder cancer]
Abdomen & chest	11	[3, 6, 9, 11, 13, 14, 16, 20, 24]	– *"I felt mad when I had pain. My pain is because of a mass in my abdomen. My abdomen is enlarged and I feel myself uncomfortably full." [9]* [Not reported]<br>– *"So I came home and there was, there was no pain really, odd, odd little bits but not, not anything and then suddenly it started to kick in my, my tummy, I got abdominal pains in my tummy". [13]* [Pancreatic cancer]

Location	Number of sources	Sources	Example supporting quotes
			– *''I cannot sleep well at night, I have an intermittent pain here (pointed to lower abdomen) and I can only sleep 3-4 hours. The pain wakes me up." [14]* [Not reported]<br>– *"What's more, it is painful in this area (pointing to right side of his abdomen) and sometimes eating can induce the pain." [14] [Not reported]*
Widespread locations	11	[2, 6, 11, 12, 14, 18, 20, 23, 27]	– *"The variation in pain can be across locations in the body, with some participants experiencing pain in multiple places." [2]* [Not reported]<br>– *One person reported that "every bone in your body is aching" and another said "even my toenails hurt." [27]* [Not reported]<br>– *"Some participants indicated excruciating pain in multiple areas of their body. They reported the pain, to some extent, had taken over their whole body." [14]* [Not reported]<br>– *"My pains have no specific location and spreads throughout the body!" [11]* [Not reported]
Joints	9	[6, 15, 20-23, 25, 27]	*"I would say the joint pain more so has been frustrating, and it has not impacted my way of thinking about taking it. My husband has said, 'You should think about whether you still want to deal with this with all the issues that you are having with it.' But I feel like that is not really a viable option, meaning that I do not think I should stop taking it because, again, the benefits outweigh the challenges." [23]* [Breast cancer]<br>– *"In some instances, the source of pain was readily apparent, such as pain in a radiation field or muscle and joint aches after a bisphosphonate treatment." [20]* [Not reported]<br>– *"They told me that they don't believe that my joint pain is from my chemo…my pain doctor told me that, he said I think it's something else…he made me feel bad…like I was making up something.'' [15]* [Not reported]<br>– *"It's just deep joint pain, it hurts. It hurt so badly that I can't function. I got to the point I could hardly move to get dressed." [25]* [B-Cell lymphoid malignancies]
Bones	8	[1, 6, 16, 19, 20, 23, 26, 27]	– *"I can't take it (the pain) any more … distending pain, feels like my bone is bursting open (covered her face with her hands)." [26]* [Breast cancer]<br>– *"As the study began, he had a significant reduction in the intensity of his metastatic bone pain with the adoption of a more effective pain management regimen." [20]* [Not reported]<br>– *"I think that the most outstanding side effect for me is joint, bone and muscle pain." [23]* [Breast cancer]<br>– *"It's very nasty. I do not like it one bit, but I do not feel I have a choice. It causes a lot of muscle and bone pain." [23]* [Breast cancer]

Location	Number of sources	Sources	Example supporting quotes
Legs and feet	7	[6, 14, 20, 23, 25, 27]	– Author commentary: Mr. T, who before his cancer diagnosis would ride his bicycle all over town, now walked with a cane because of the discomfort from the large tumor on his left thigh. *[27]* [Not reported]<br>– *"I mean a lot of pain and the pain ranges from my thigh, to my knee, to my ankle, and it is a very aggravating, sharp pain that make you wish you didn't have it". [27]* [Not reported]<br>– *"I got a massage once or twice to help with the feet, but that's just temporary. That's just a temporary relief." [23]* [Breast cancer]
Head	6	[6, 9, 11, 20, 23, 25]	– *"Well, there were times when I just had a headache constant through the whole day. Not like a migraine, but just like that headache that just stays there. I was taking pain medications, but it was something that was there the whole time." [6]* [Acute myeloid leukemia]<br>– *"My headache got so severe that I imagined them cutting my head off and throwing me on the ground." [11]* [Not reported]<br>– *"I got these intense pains in my head, stabbing, shooting pains." [25]* [B-Cell lymphoid leukemia]
Knees	5	[6, 20, 21, 25, 27]	– *"It's just deep joint pain, it hurts. It hurt so badly that I can't function. I got to the point I could hardly move to get dressed. I had to have my husband help dress me, because I couldn't move my shoulder joints, or my knees, my ankles were in terrible pain. It affected my walking." [25]* [B-Cell lymphoid malignancies]<br>– Author commentary: Five women reported joint pain, one describing it as if someone had "hit [her] in the kneecap with a hammer. *[21]* [Breast cancer]
Neck & shoulder	4	[7, 11, 25]	– *"Now my pain is terrible and affects my back and neck. This bothers me considerably. The pain begins in the middle of my back and spreads to the shoulder, and then to the fingertips." [11]* [Not reported]
Urogenital/ rectal	4	[5, 20, 27]	– *"It burned every time I had to pee…but two or three days after you get normal again". [5]* [Cervical cancer]
Breast	3	[11, 21, 27]	– Author commentary: Most of our participants reported their pain as widespread and radiated into different parts of their body. The body parts of participants that involved in pain were breast, chest, neck, back, waist, arm, abdomen, ribs, and heart. *[11]* [Not reported]
Muscles	3	[10, 20, 23]	– *"It's very nasty. I do not like it one bit, but I do not feel I have a choice. It causes a lot of muscle and bone pain. I've already sort of not been a great sleeper since going through menopause, and it's just made that worse. I feel I'm crabbier, probably because I do not sleep as well, and I'm often uncomfortable. I do not really feel like I have a choice, so on we go". [23]* [Breast cancer]

Location	Number of sources	Sources	Example supporting quotes
Nerve pain	3	[10, 20, 23]	– Author commentary: Thereafter, she had a brief but severe muscle spasm with a stressful event, pain in her Achilles tendon, and what she described as sciatic nerve pain. *[20]* [Not reported]
Skin	3	[4, 20, 27]	– Author commentary: Three women spoke of the pain from skin burns that they received from radiation treatment for their cancers (lung, breast, or cervical), and each speculated that they had received too much radiation. *[27]* [Not reported]
Arms	3	[11, 20]	– Author commentary: Nancy had pain in one arm throughout the study, associated with a tumor in her brachial plexus and lymphedema. *[20]* [Not reported]
Groin and pelvis	3	[16, 20]	– *"So the pain is sharp and kind of a sharp throbbing type, kind of constant pain with kind of sharp nuisances to it in my groin area." [20]* [Not reported]
Hands	3	[20]	– Author commentary: Valerie described pain from bone metastases and chemotherapy-related neuropathic pain in her hands throughout the 10 weeks. *[20]* [Not reported]
Ribs	2	[11, 20]	– Author commentary: She experienced waxing-and-waning pain in her back, ribs, and sternum with each chemotherapy infusion. *[20]* [Not reported]
Hip	2	[20, 22]	– *"And then with my back and hip, there are very, very sharp pains, and that's where I say a pinched-nerve–type pain." [20]* [Not reported]
Throat and mouth	1	[20]	– *"It's all about where your pain is, and this last couple of weeks it's been the esophagus." [20]* [Not reported]
Scar tissue	1	[10, 20, 23]	– Author commentary: Thereafter, she had a brief but severe muscle spasm with a stressful event, pain in her Achilles tendon, and what she described as sciatic nerve pain. *[10, 20, 23]* [Not reported]

Impacts

Physiological

Table S10 provides an overview of the physiological impacts of cancer-related pain, as reported in the literature.

Table S10. Physiological impacts of pain reported in the qualitative literature.

Impact	Number of sources	Sources	Example supporting quotes
Fatigue	6	[2, 9, 12, 14-16]	*"I felt myself in depressed mood, fatigue, dizziness, all make me miserable … so I do not want to do anything, close the curtains and stay in bed for many hours … I even do not want to see my grandchild who is my favorite person …" [9]* [Not reported]<br>– *"I feel tiredness and powerlessness to go to work and do something, all the tasks are waiting for me …. Pain restricts not only daily life, it disturbs working life and causes economic problems." [9]* [Not reported]
Weakness	5	[9, 11, 14, 23, 26]	*"I am unable to sit down. I cannot adhere to the proper greeting etiquette. To the point where my arms and legs are weak and listless." [11]* [Not reported]<br>– *''When breakthrough pain comes, I sweat all over…so all my clothes are soaking wet and I suffer terribly from being so weak and tired.'' [14]* [Not reported]
Lack of appetite	4	[9, 13, 24, 26]	– *"I don't have any appetite when in pain, but if my son comes along with me, I will eat more." [26]* [Non-Hodgkin's lymphoma]
Sweating or shivering	2	[14, 19]	– *''When breakthrough pain comes, I sweat all over ...so all my clothes are soaking wet and I suffer terribly from being so weak and tired.'' [14]* [Not reported]
Itching	2	[10, 19]	– Author commentary: Furthermore, participants described altered sensations, about half of the women experienced numbness and uncomfortable sensations (stinging, tingling), and a few experienced hypersensitivity or itching. *[10]* [Not reported]
Breathing difficulties	2	[3, 9]	– *"It disturbed all my life, I could not walk, breathe, I could not sleep …. Even my posture, I could not sit comfortably…" [9]* [Not reported]

Impact	Number of sources	Sources	Example supporting quotes
Dizziness	2	[9, 14]	– *"When my body is in pain, I would profusely sweat over my forehead. I cannot sit up without help…and I feel very dizzy so I cannot walk by myself.'' [14]* [Not reported]
Exhaustion	2	[12, 15]	– Author commentary: He was oxygen dependent and was not taking his prescribed pain medication for fear it would stop his breathing. He shared that he only slept 2 hours each night because of his severe pain. Lack of sleep and fatigue from the unrelenting pain and COPD left him exhausted. *[15]* [Not reported]

Physical

Table S11 provides an overview of the impacts of cancer-related pain on physical functioning, as reported in the literature.

Table S11. Physical functioning impacts reported in the qualitative literature.

Impact on/difficulty with	Number of sources	Sources	Example supporting quotes
Walking	11	[3, 6, 9, 14, 16, 18, 24-28]	– *"I couldn't walk. I couldn't move my limbs…because the pain would be great. So that morphine has saved me, to move about." [28]* [Not reported]<br>– *''I cannot walk, breakthrough pain can occur even when I am just sitting up or eating and I cannot do anything without assistance." [14]* [Not reported]<br>– *"It disturbed all my life, I could not walk, breathe, I could not sleep…. Even my posture, I could not sit comfortably…" [9]* [Not reported]<br>– *"I just lie still in bed. It hurts too much to move and walk (sighed)." [26]* [Lung cancer]
Exercise	10	[1, 3, 7, 10, 14, 16, 18, 26-28]	– *"My shoulder hurts all the time so I don't exercise, I can't bring myself to go out and exercise and that makes me feel, you know, it sort of makes me tired mentally." [7]* [Not reported]<br>– *"I can't do anything that I used to do in terms of walking, running. I just have to do it in a different way." [28]* [Not reported]<br>– *"When I make certain movements, it pulls. The fact that I'm limited in my actions, which I did not have before... for example when I went for a swim...*

			and that's actually also the case with skiing." [10] [Breast cancer]
Range of motion	4	[10, 15, 17, 26]	– *"Really, to just reach out like that is very difficult and very painful and it doesn't go so well either. So in everyday life you come across a whole lot of things, in which you can actually not use that left arm in the same way as the right one."[10]* [Breast cancer]
Getting out of bed	3	[9, 11, 26]	– *"I just lie still in bed. It hurts too much to move and walk (sighed)." [26]* [Lung cancer]
Limited movement/ unable to move	3	[15, 26, 28]	– *''It was unbearable... it was excruciating…where I couldn't move.'' [15]* [Diagnosis not reported]
Sitting	3	[9, 11, 14]	– *"I can neither sit up, nor lay down, what a disaster." [14]* [Not reported]
Transitional movements	1	[14]	– *"Sometimes the pain even made sitting down, getting up, and turning over become extremely difficult." [14]* [Not reported]

Emotional

Table S12 provides an overview of the impacts of cancer-related pain on emotional wellbeing, as reported in the literature.

Table S12. Emotional wellbeing impacts of pain within the qualitative literature.

Impact on/feeling	Number of sources	Sources	Example supporting quotes
Fear	15	[1, 2, 5-8, 10, 12-14, 19, 22, 23, 26, 27]	– *"I'm scared. A woman said it's very painful, even worse than giving birth they said!" [5]* [Cervical cancer]<br>– *''I am scared because I do not know when it (breakthrough pain) will attack me next time so I am nervous all day long." [14]* [Not reported]<br>– *"I called the hospital and then I finally started taking it. I was really frightened, I did not want any more pain. I'm very sensitive to pain in my hands and my feet and I just did not want that sort of thing." [23]* [Breast cancer]<br>– *"The pain is always there in the middle. It is all black and spreading out. I am always afraid that it will come back and this time it will get me. There is no escape from this blackness." [12]* [Diagnosis not reported]
Sadness/ depression	9	[3, 9-11, 14, 18, 23, 26, 27]	– *"I'm still sad and not as happy as I was before. As a result of this pain, I'm not happy at all; nothing makes me happy these days." [11]* [Not reported]<br>– *''Pain is a life-threatening symptom for me. It makes my life miserable, I no longer can do things like a normal person [sighed]. I can't even take a walk outside the ward because*

Impact on/feeling	Number of sources	Sources	Example supporting quotes
			I don't know when the pain will attack." [14] [Not reported]<br>– *"I feel stigma, depressed. I have been talking less now and am unwilling to communicate with those around me (voice grew weaker)." [26]* [Breast cancer]<br>– *Author commentary:* In interviews, about half of the women and half of the men in the sample talked of feeling depressed, although the men did not speak about it in depth. *[27]* [Not reported]
Anxiety/ worry	9	[2, 5, 6, 8, 9, 14, 18, 21, 27]	– Author commentary: Pain was not only expressed as a physical symptom but also as an emotional one that included depressed mood, worry, anxiety, and fear. *[27]* [Not reported]<br>– *"I couldn't sleep… I kept thinking of the pain I'm going to go through". [5]* [Cervical cancer]<br>– Author commentary: Due to the unexpected onset and uncertainty about the cause, participants identified "an element of anxiety" associated with their pain and concern that it signalled a return or metastasis of the cancer. *[21]* [Breast cancer]<br>– *"The kind of pain you mostly have, that wor-ration, that worry is what kill people too… so much pain from dealing with what you're going through, and it adds on to what you can't do nothing about, and then it becomes a problem, an agony". [27]* [Not reported]
Hopelessness	8	[9, 12, 14, 15, 17, 23, 26, 27]	– *"All I want to do is to die. It hurts so much that no self-suggestions are helpful. I've collapsed...the pain is killing me, I want to give up." [26]* [Colon and rectum cancer]<br>– *No plan, even if there is, it can't be completed. On the contrary, it will be a burden to my son and daughter." [26]* [Myeloma]<br>– *"Both of these women expressed sadness and hopelessness over the inability to control their pain." [27]* [Not reported]<br>– *"Pain is a bad experience. I could not think pleasant things when I had pain, ….feel myself helpless and hopeless …. " [9]* [Not reported]
Anger/ frustration	8	[9-11, 14, 18, 23, 26, 27]	– *"Pain makes me miserable and angry. Everything seems a huge problem, when I had pain." [9]* [Not reported]<br>– *''the pain tortures me so much. When I am in pain, I would refuse to talk with anyone or throw a temper. I am fed up with this.'' [14]* [Not reported]<br>– *"When it hurts, it's hard not to get angry. I can't control my temper and I'm getting more impatient than before. I*

Impact on/feeling	Number of sources	Sources	Example supporting quotes
			have a feeling that I'm getting worse …" [26] [Liposarcoma]<br>– *"One day I was with my neurologist when she was talking about the treatment plan […], and what I was trying to describe to her was not matching that. And I just broke down in frustration and cried, and I said you're not listening to me. I'm trying to tell you this is what's hurting. This is the way it feels. And then, she stopped and she actually listened and that's when she began to put me on the journey of physical therapy and now, pain management." [18]* [Breast cancer]
Suicidal or death-related thoughts	5	[11, 14, 15, 26, 27]	– *"I feel like it's better to receive euthanasia when pain is extremely intense. It's meaningless to live for extra years. I prefer to die rather than live with endless pain." [26]* [Lung cancer]<br>– *''It's horrible. This unbearable pain experience makes me can do nothing but cry, and I want to die rather than continue suffering from this pain. I cannot bear it.'' [14]* [Not reported]
Helplessness	5	[3, 5, 8, 21, 23]	– Author commentary: While participants suffered various forms of distress during the treatment process, most indicated that they had no alternative. A sense of helplessness was reflected by their acceptance that nothing could be performed to relieve the symptoms, and that they merely hoped the treatment would be finished as quickly as possible. *[5]* [Cervical cancer]<br>– Author commentary: Some women concluded that pain was just an inevitable outcome of being treated for breast cancer. Feeling that nothing could be done to help them, many participants kept the pain to themselves and coped as best they could.*[21]* [Breast cancer]
Loss of self/ autonomy	5	[3, 10, 11, 18, 28]	– *"Before I became ill, I was really strong. I had many plans for my life. I used to exercise and was in great shape, but… But what now? My life has been entirely upended. I look at the people walking outside and think, "God, we were once like them." That is, I can return to the same person I was previously." [11]* [Not reported]<br>– *"This is why I was getting depressed, I think, too. I'm one of those – I don't sit still. I don't relax. And that's just who I am. And that's fine. That's just who I am. And It wasn't doing a lot of things that I loved and it made me really – it just made it harder." [18]* [Breast cancer]

Impact on/feeling	Number of sources	Sources	Example supporting quotes
Isolated	5	[9, 12, 22, 26, 28]	– *"I feel stigma, depressed. I have been talking less now and am unwilling to communicate with those around me (voice grew weaker)." [26]* [Breast cancer]<br>– *"I felt myself in depressed mood, fatigue, dizziness, all make me miserable … so I do not want to do anything, close the curtains and stay in bed for many hours … I even do not want to see my grandchild who is my favorite person …" [9]* [Not reported]
Irritability	4	[9, 11, 23, 26]	– *"I had a good temper before, but now I get much more irritable when in pain." [26]* [Breast cancer]
Fatalism	4	[3, 9, 14, 17]	– *Author commentary:* Patients in this study expressed some fatalistic beliefs that there was nothing they could do to prevent cancer pain nor to avoid death from cancer or that they would rather die to avoid the pain. They might expect pain was inevitable in cancer so it ought to be ; endured. *[14]* [Not reported]
Distress	4	[13, 15, 18, 28]	– Author commentary: The intensity and duration of pain, and lack of effective strategies for reducing pain were frequently mentioned as contributing to distress, anxiety, and depression. *[18]* [Not reported]
Embarrassment or shame	4	[3, 7, 11, 13]	– *"I'm not independent, I can't wash, you know, not properly, well, I'm just starting to be able to, to do that, but my wife had to wash me. That is significant you know, for anybody I think, because even though it's your nearest and dearest, to have to strip off in the kitchen and be washed, like a little child, you know, it can be a bit embarrassing." [3]* [Not reported]
Resilience	3	[3, 5, 14]	– *"The breakthrough pain is a normally disease itself; sometimes I could bear it and no needed to report it. And you know, it works well to my pain management. The repeated suffering from breakthrough pain makes me stronger." [14]* [Not reported]
Low self-esteem or worthlessness	2	[11, 26]	– *Although shedding tears and complaining about her worthlessness, the patient continued his speech as follows: "My life has no value anymore. I can't do anything. I lost my dignity as a human being." [11]* [Not reported]

Activities of Daily Living (ADLs)

Table S13 provides an overview of the impacts of cancer-related pain on ADLs, as reported in the literature.

Table S13. Activity of daily living impacts reported in the qualitative literature.

Impact on/difficulty with	Number of sources	Sources	Example supporting quotes
Household tasks	8	[2, 9, 10, 15, 18, 20, 26, 27]	– *"I just want, that's what I want, I just want normality, but the normality's gone, there's no normality anymore, unless I'm on my own, but even then I can't do, I look at the house and I think god if I wasn't ill I'd have this decorated and all sorts done by now, and it's horrible, because I know as soon as do something I'm going to be in agony." [2]* [Breast cancer]<br>– Author commentary: They could not manage daily household chores and became dependent on other family members. *[9]* [Not reported]<br>– *"I used to do housework, but now I contribute nothing to the family except for imposing troubles and burdens." [26]* [Breast cancer]<br>– Author commentary: Several reported no longer being able to work or complete daily household tasks and a few had issues with basic functioning including eating, walking or sleeping. *[18]* [Not reported]
Leisure activities/ hobbies	8	[1, 3, 10, 16, 18, 20, 26, 27]	– *"The pain really restricted what I was doing so I, I didn't want to go out particularly, I didn't want to sing in the choir, y'know, those sort of things, it just, it just knocked me, knocked me sideways really." [3]* [Not reported]<br>– *"I have to abandon my square dancing hobby." and "Regrettably, I'm unable to continue my outdoor photography." [26]* [Breast cancer]<br>– *"I think it has affected me socially…walking…I mean we used to walk right down to our friends every Saturday night, and I couldn't walk round there now…I'm just trying to think of the things I used to do. Rambling and bowling and all this that and the other" [3]* [Not reported]
Independence	6	[3, 9, 11, 14, 15, 27]	– *"It's (the pain) caused me to be less independent and self-sufficient. I'm having to depend on others to do certain things 'Cause I cannot move, function, you know, without it…Like I was saying about the pain medicine, with it, I function, without it, I don't function." [27]* [Not reported]<br>– Author commentary: Perceived loss of independence as well as fear of burdening one's family were key underlying issues as participants struggled with cancer pain. Burden was expressed in a variety of ways, from fear of emotionally burdening others, to the physical burdens placed on family members when mobility and self-care is compromised by pain. *[3]* [Not reported]<br>– Author commentary: Participants described how pain robbed them of their independence. One participant, who had been a nurse for many years and had previously enjoyed and taken pride in her work, explained that pain had robbed her of a purpose in life because she could no longer work or take

Impact on/difficulty with	Number of sources	Sources	Example supporting quotes
			control over the parts of her life that gave her meaning. *[27]* [Not reported]
Eating	4	[11, 14, 18, 27]	– Author commentary: Participants reported that physical pain regularly interacted with other symptoms and sometimes limited activities. One participant said he could not eat, sleep, or walk when he had pain.*[27]* [Not reported]
Self-care	4	[3, 15, 25, 26]	– *"It's just deep joint pain, it hurts. It hurt so badly that I can't function. I got to the point I could hardly move to get dressed. I had to have my husband help dress me, because I couldn't move my shoulder joints, or my knees, my ankles were in terrible pain. It affected my walking."[25]* [B-Cell lymphoid malignancies]
Caring for others	2	[6, 11]	– *"One of the female participants stated: "I feel bad when I am in too much pain and I ask my son or spouse for help. I cannot feed my son, meet his needs, or be a good mother. I cannot fulfill responsibilities to my family. I'm still ashamed."[11]* [Not reported]
Staying at home	2	[3, 22]	– *"I'd have loved to have gone to Russia, but now my life, my world, is an electric recliner"[3]* [Not reported]

Sleep

Table S14 provides an overview of the impacts of cancer-related pain on sleep, as reported in the literature.

Table S14. Negative sleep impacts described in the qualitative literature.

Impact	Number of sources	Sources	Example supporting quotes
Duration of sleep/ awakening due to pain	7	[9, 14, 15, 19, 22, 26, 27]	– *"I had difficulty falling asleep and woke up almost five times a night because of pain …" [9]* [Not reported]<br>– *"Participants who reported an inability to sleep attributed it to being in pain; others reported being awakened because of pain." [27]* [Not reported]<br>– *"I have been waking up five to six times at night. I have been sitting on the toilet for comfort. My yelling in pain can be heard by the whole ward (laughed with embarrassment)." [26]* [Lung cancer]
Sleep quality and efficiency	5	[9, 14, 22-24]	– *"It's very nasty. I do not like it one bit, but I do not feel I have a choice. It causes a lot of muscle and bone pain. I've already sort of not been a great sleeper since going through menopause, and it's just made that worse. I feel I'm crabbier, probably because I do not sleep as well, and I'm often uncomfortable. I do not really feel like I have a choice, so on we go." [23]* [Breast cancer]<br>– *"The symptom that bothered me the most after the operation was wound pain. Although I used the analgesic pump and received a*

			painkiller, I still felt pain in my chest; thus, I did not experience cough. I did not have much of an appetite and could not sleep well because of pain. The pain is a major problem for me." [24] [Lung cancer]
Initiation/ difficulty falling asleep	5	[9, 14, 17, 24, 27]	– *"I went to bed at 10 PM but I couldn't fall asleep because the unexpected pain." [14]* [Not reported]<br>– *"When asked if she was still taking the pain medicine, she told us that the pain was "so overwhelming" that she had to take the pain medication. If she did not take the pain medication, her pain kept her from sleeping." [24]* [Lung cancer]

Social Functioning and Relationships

Table S15 provides an overview of the impacts of cancer-related pain on social activities, as reported in the literature.

Table S15. Social impacts of pain reported in the qualitative literature.

Impact	Number of sources	Sources	Example supporting quotes
Participation in social events	5	[3, 9, 10, 16, 28]	– *" …. Because of pain, I could not go out, do even an easy household or visit any neighbour … I am in social isolation, not want to hear anyone's voice …" [9]* [Not reported]<br>– *"I think it has affected me socially…walking…I mean we used to walk right down to our friends every Saturday night, and I couldn't walk round there now…I'm just trying to think of the things I used to do. Rambling and bowling and all this that and the other." [3]* [Not reported]
Family role/ relationships	3	[11, 16, 26]	– *"I feel bad when I am in too much pain and I ask my son or spouse for help. I cannot feed my son, meet his needs, or be a good mother. I cannot fulfil responsibilities to my family. I'm still ashamed." [11]* [Not reported]
Romantic relationships (dynamics and intimacy)	2	[3, 16]	– *"I'm not independent, I can't wash, you know, not properly, well, I'm just starting to be able to, to do that, but my wife had to wash me. That is significant you know, for anybody I think, because even though it's your nearest and dearest, to have to strip off in the kitchen and be washed, like a little child, you know, it can be a bit embarrassing." [3]* [Not reported]

Work and Financial

Table S16 provides an overview of the impacts of cancer-related pain on work and finance, as reported in the literature.

Table S16. Work and financial impacts described in the qualitative literature.

Impact	Number of sources	Sources	Example supporting quotes
Disruptions to working life	6	[9, 10, 18, 23, 26, 28]	– *"I feel tiredness and powerlessness to go to work and do something, all the tasks are waiting for me …. Pain restricts not only daily life, it disturbs working life and causes economic problems." [9] [Not reported]*<br>– *'It was really painful and causing cramping when I'm sleeping and when I'm at work." [26] [Breast cancer]*<br>– *"It took a minute for me to express these things to other people other than my family, because I really wanted to go through this kind of independently, but as time went on, I was, you know, having to stay off work, and I had to share it with more and more people." [28] [Not reported]*
Unable to work	5	[11, 18, 26-28]	– *Author commentary: Many had to make major life adjustments because of pain, especially in the area of employment, either by reducing working hours or quitting. [27] [Not reported]*<br>– *Author commentary: The story of Ms. D illustrated how her life changed because of neuropathic pain. She described having to quit the job she loved. Her job required her to drive long distances, and because of the numbness and discomfort in her feet, she had to quit. "The chemo tore up the nerves in my leg and my feet. And my right foot, you know, you drive with your right foot. My right foot, it feels like, a bunch of sand is up under here, and my toes feel like they was swollen, and I hit on the brakes, actually it didn't feel like I was stepping on the brakes. [27] [Not reported]*<br>– *"It has had a huge impact on my work. I can't continue my original work anymore." [26] [Breast cancer]*
Loss of earnings	2	[9, 11]	– *"I feel tiredness and powerlessness to go to work and do something, all the tasks are waiting for me …. Pain restricts not only daily life, it disturbs working life and causes economic problems." [9] [Not reported]*<br>– *Author commentary: Some participants stated that they are unable to work due to cancer and related pain, causing financial difficulties for their families. [11]* [Not reported]

Coping Strategies

Table S17 presents the coping strategies for cancer-related pain identified within the qualitative literature.

Table S17. Coping strategies described in the qualitative literature.

Coping strategy	Number of sources	Sources	Example supporting quotes
Pain medication	19	[1, 2, 5, 7-9, 11, 13-17, 19-21, 23, 24, 26, 28]	– *"When I feel pain, I take medication prescribed by my doctor. As my pain is about to begin, I will immediately take the oxycodone that he recommended." [11]* [Not reported]<br>– Author commentary: Despite his original reservations, patient three reported a good effect from his morphine saying his doctor 'put me on it and it certainly helped, you know.' *[13]* [Not reported]<br>– *"What has helped me the most was that I have learned to take the analgesics around the clock." [7]* [Not reported]<br>– *"I couldn't walk. I couldn't move my limbs…because the pain would be great. So that morphine has saved me, to move about." [28]* [Not reported]<br>– *"I believe that it's a necessary evil. Because I need to take it in order for me not to be in pain. I need it in order to function properly. 'Cause if I don't take my pain medication, I can't do, I can't move, I'm sick, I, I'm in so much pain that I, I literally cannot do anything." [28]* [Not reported]
Stoicism and resilience	13	[1, 3, 7, 9, 11, 14, 15, 17-19, 21, 23, 28]	– *"Don't let the pain define you, and if you come upon, you know, a little challenge, just back off and adjust. Back off and adjust, and then you will find the answers." [28]* [Not reported]<br>– Author commentary: For some, using inner strengths was expressed as a desire to actively fight pain, with one participant stating, "my attitude towards it is, if I could strangle the bugger, I would. *[3]* [Not reported]<br>– *"Some patients shared that it was ''normal'' to have pain when you have cancer and that you just have to be strong and endure the pain." [7]* [Not reported]<br>– *"I hate that anybody has to go through pain like I have to go through it, and I'm sure there's people out there that's in worse pain than I am. So, I don't try to complain any." [18]* [Lung cancer]
Concealment of pain	10	[1-3, 13-15, 18, 19, 21, 28]	– *"I've never believed in showing pain to other people, y'know, take this to extremes now, say I cut my finger off, I'd go ow, and I'd wrap it up, and that would be it. I wouldn't go ooh, ooh. I don't like doing that, and I don't like people who do it." [3]* [Not reported]

Coping strategy	Number of sources	Sources	Example supporting quotes
			– *"I try to hide a lot of the feelings I was getting cause I didn't want to be a burden to my family, so I sort of kept it a secret to some extent, and tried to carry on as normal y'know." [3]* [Not reported]<br>– *"I wouldn't tell anybody…it was hard to feel the way…but I didn't tell my kids that it was that bad." [15]* [Not reported]<br>– Author commentary: Participants tended to either keep the pain experience private or share it with only a tight circle of family and friends. Some felt it was necessary to keep their suffering to themselves; it was a private experience that was never shared with most friends, neighbours, or coworkers. *[28]* [Not reported]
Self-management	9	[1-3, 7, 15, 17, 19, 23, 28]	– *"I've medicines upstairs and medicine downstairs so that if I get stuck on one level at least I've only got to get to the front room cause it's on the bottom window, so if I'm upstairs I only have to get to the bedroom cause it's on the dressing table top in there, so I know I've not got very far to go to get medicine." [2]* [Breast cancer]<br>– *"I've just developed a system where I take my medication at certain times and I don't wait for the pain to start and then take the medication. I take it before it starts, and that's been the secret of it working for me." [3]* [Not reported]<br>– Author commentary: However, other participants appeared not to view medical professionals as holding exclusive knowledge about managing pain and they therefore self-determined their own medication regimes according to their own requirements. Participants often perceived themselves as experts themselves in regard to their pain and their needs. *[3]* [Not reported]
Positional changes and stretching	8	[9, 13, 14, 19-21, 23, 26]	– *"When I have pain, I walk along the corridor at home, do some stretching exercises with my spring tool." [9]* [Not reported]<br>– *''It is painful for me to turn to the right side. When I am in pain, I try to find the best position to alleviate the pain as much as possible. Then I remember to keep at this position to avoid pain in the future.'' [14]* [Not reported]<br>– *"I often lie in bed and do Baduanjin (traditional Chinese exercise) and breathe deeply. I practice when I wake up in the evening or before dawn with pain." [26]* [Colon and rectum cancer]<br>– Author commentary: Executing movements, adopting physical positions and getting involved in activities in order not to think about the pain were found to be some of the coping strategies devised and

Coping strategy	Number of sources	Sources	Example supporting quotes
			described by patients to cope with painful sensations. *[19]* [Not reported]
Spirituality/prayer	8	[3, 5, 9, 11, 15, 17, 26, 28]	– *"The morphine didn't stop it, it only minimized it… God helps me get through the pain. And that's why I lean on him right to this day. Because God, I'm able to manage." [17]* [Multiple myeloma]<br>– *"… I pray to God for my healing and namaz (an Islamic pray ritual, performed five times a day) in order to manage with the pain." [9]* [Not reported]<br>– *"My daughter went to the temple and asked for a safety symbol for me (placed a hand over it on her chest), hoping to bless me to allow escape from the suffering. She also downloaded some Buddhist sutras and music, which I stored in my mobile phone. It works when I'm restless with pain." [26]* [Breast cancer]<br>– *"Just praying about my situation and knowing that, you know, God has my back and problems. That's helped. That's the number 1 thing that's helped — God." [28]* [Not reported]
Massage	7	[11, 13, 14, 17, 21, 23, 27]	– *"I got a massage once or twice to help with the feet, but that's just temporary. That's just a temporary relief." [23]* [Breast cancer]<br>– *"Ms. K expressed being tired of the pain and tired of taking medication. She hurt throughout the interview. Her roommate sat next to her during the interview and massaged her legs. She spoke of giving up." [27]* [Not reported]<br>– Author commentary: This strategy included physical interventions (touching, massaging, and positioning the painful area, and heat and cold therapy), self-medication (opium (Taryak in Persian) and tobacco use, herbal pain treatments, and adhering to a pain relieving diet) *[11]* [Not reported]
Alternative medicine	7	[7, 9, 11, 14, 17, 21, 23]	– *"I had an ancient therapy which is bloodletting with cupping, it is popular nowadays. I also received some physiotherapy but did not use any other products." [9] [Not reported]*<br>– *"I used to have a lot of body aches, but then I went for alternative medicine." [23] [Breast cancer]*<br>– Author commentary: Some patients also employed alternative treatments such as massage, acupuncture, and other nonpharmacological methods to self-manage breakthrough pain. *[14]* [Not reported]

Coping strategy	Number of sources	Sources	Example supporting quotes
Optimism and positivity	6	[3, 5, 11, 13, 18, 23]	– *"You've got to have hope haven't you? Y'know, you've got to, you've always got to think well there's an answer around the corner to this problem y'know. I haven't found it yet, well, I must've done because I cope. I don't know what it is, why I cope, I'm not brave, I'm nothing special, but I just try and not let it get me down." [3]* [Not reported]<br>– *"That's another key factor, being positive y'know. I never look on the negative side, and again I think that's really important, for everyone to adopt a positive attitude." [3]* [Not reported]<br>– *"I only take two Paracetamol in the morning and, and that sees me through all whole day, whether it's a mindset or not, I don't know, because I am actually trying to be very positive about this and, as far as I'm concerned, I haven't got it and I'm treating life as I did in the past, so whether it's, as I say." [13]* [Pancreatic cancer]
Heat or cold	6	[2, 9, 13, 14, 17, 21]	– *… I take a hot bath … bathing relaxes me so much, feel myself better …" [9]* [Not reported]<br>– *''I would apply hot-water bag or towel on my abdomen to ease the pain.'' [14]* [Not reported]<br>– Author commentary: Most women adopted a trial and error approach to pain management, using a wide range of strategies, including: "massage, the TENS [Transcutaneous electrical nerve stimulation] machine, ice packs, heat packs, [prescription narcotics], anti-depressants… *[21]* [Breast cancer]
Light physical activity	6	[3, 9, 19, 21, 23, 26]	– *"I often lie in bed and do Baduanjin (traditional Chinese exercise) and breathe deeply. I practice when I wake up in the evening or before dawn with pain." [26]* [Colon and rectum cancer]<br>– *… I walked around at home, watched TV and slept …. not to concentrate on my pain …" [9]* [Not reported]
Knowledge of pain	5	[3, 7, 15, 17, 21]	– *"I think the first thing that you need to know is what's caused the pain, you need to know that, so you can understand what the pain is, and I think if you understand what the pain is, you can cope with it better." [3]* [Not reported]<br>– *"I was scared taking my medicines…I really was scared. If I hadn't met you…and I just went to the doctor and he said, 'just take the medicine' and think everything will work out all right…and not have some intricate knowledge of what's going on… I'd still be in a lot of pain.'' [15]* [Not reported]

Coping strategy	Number of sources	Sources	Example supporting quotes
Distraction	5	[1, 3, 9, 11, 26]	– *"While undergoing chemotherapy, I completed my high school graduation and am currently enrolled in an undergraduate program. When I am preoccupied with something else, I forget my pain." [11]* [Not reported]<br>– *"I have to focus on something y'see, all the time, and somebody said this to me about getting better, do the things you normally do…I'm also focusing on picking me brambles and doing the garden. I can't just sit y'see, even though I'm poorly I have to try and do something." [3]* [Not reported]
Dietary changes	3	[11, 14, 21]	– Author commentary: Most women adopted a trial and error approach to pain management, using a wide range of strategies, including: "massage, the TENS [Transcutaneous electrical nerve stimulation] machine, ice packs, heat packs, [prescription narcotics], anti-depressants…", as well as resistance training, weights, hydrotherapy, Tai Chi, stretching, meditation and dietary changes. *[21]* [Breast cancer]
Antidepressants	3	[9, 21, 23]	– *"I take my antidepressant drug, it makes me feel better. I do needlework like knitting as a therapy, spend a lot of time in the garden … I forget my illness while digging the soil. Now, I am planning what to plant for this year." [9]* [Not reported]
Sleep	2	[2, 9]	– *"I usually take an analgesic and have a long sleep all day." [9]* [Not reported]
Treatment break/change	2	[21, 23]	– *"He [oncologist] said, "If this does not work then we could switch to the letrozole," and it's been helping. It was mostly the leg pain that I had a little bit of issue with all through my treatment, especially with the Neulasta and that whole thing. So, he said, "All right, well, let us try it." And honestly, it was pretty quick that I realized it was a much better fit for me." [23]* [Breast cancer]
**Sources of support**			
Family/partners	6	[1, 3, 17, 23, 25, 28]	– *"Bless my wife, she's a very patient and good lady and she would always just stop everything and just sit with me…she has been excellent at making sure I don't lose sight of reality." [3]* [Not reported]<br>– *"the main thing is my girlfriend, she's the great salvation, she's the angel…we all need to love somebody." [3]* [Not reported]
Healthcare professionals	6	[2, 3, 8, 15, 17, 23]	– Author commentary*:* Support was also experienced in terms of having a "back-up team," knowing that

Coping strategy	Number of sources	Sources	Example supporting quotes
			medical help was accessible. The knowledge that the hospital team was available, if the situation deteriorated and circumstances rendered it necessary, appeared to enable participants to stay at home, comfortable in the knowledge that help could be accessed. *[3]* [Not reported]<br>– *"One time I came to see the doctor, and he saw my face and he's like, 'You're not taking the painkillers, are you?' And I am like, 'No.' He's like, 'Why are you suffering?' I'm like, 'Well I've heard that you get addicted to painkillers and I don't wanna be that person'. And he's like, 'I will tell you if you can get addicted to painkillers. So take the painkillers. You're not going to be on them for life, so you're gonna be okay.' So when he told me that I started taking them…After that I was a lot better." [17]* [Multiple myeloma]
Wider support networks	5	[1, 3, 11, 21, 23]	– *"I found a website with women from all over the world complaining about exactly the same pain as I had so I felt, oh… I'm not going [crazy]" [21]* [Breast cancer]<br>– *"I try to spend my day with people who make me feel good. I have friends who give me a lot of energy. In my opinion, cancer cells multiply hundreds of time faster if I am not happy for a moment. The more you laugh, the more cancer is suppressed." [11]* [Not reported]
Hospice or pain clinic	3	[2, 9, 21]	– *"… when you're talking with Macmillan's it's just like talking about any other thing and it's just like it's just an issue, just, and it's no different to a bag of fish and chips, really, realistically, it's just in simple terms isn't it, this is what we want to talk about, pain, and if you're in pain you do want to talk about it to somebody don't you, but they're far superior to GPs." [2]* [Prostate cancer]

Unmet Needs

Table S18 presents the unmet treatment needs identified within the qualitative literature.

Table S18. Unmet treatment needs within the qualitative literature.

Unmet treatment need	Number of sources	Sources	Example supporting quotes
Communication with healthcare providers	12	[1-3, 7-9, 11, 14, 18, 21, 23, 28]	– *"When I told the oncologist after three weeks he said 'No, it is too early for you to be feeling that sort of pain." [21]* [Breast cancer]<br>– Author commentary: Participants believed that most nurses and doctors are the busiest people and are too busy to handle their breakthrough pain. "I know they are very busy. I can understand that. There are only about 15 physicians in this 180 beds department which are usually fully occupied. So in most cases. when the pain comes, I just keep quiet and don't want to bother them. *[14]* [Not reported]<br>– *"I did not feel welcome to ask for anything unnecessarily. We have an extremely ill patient to take care of, so I felt a bit snubbed. I then watched a film, but it was not effective. I lay for five hours and had terrible pain without a second sleep and then five hours is a long time. You then concentrate on surviving." [8]* [Pancreatic cancer]<br>– Author commentary: The majority described feeling, at times, a disconnect between their own chronic pain experiences, with pain fundamentally altering their day-to-day lives, and limited communication with their providers around pain management. When they did communicate their pain to providers, participants described often feeling misunderstood. *[18]* [Not reported]
Efficacy of pain medication	11	[2, 5, 8, 11, 13, 14, 17, 21, 24, 26, 28]	– *"Eish! I feel so very painful, that pill and injection isn't strong enough…" [5]* [Cervical cancer]<br>– *"If I take it as the nurse said, then I have to live through the terrible pain without any analgesics on the last day… this tells me that there is no effective method to control my pain." [14]* [Not reported]<br>– *"All I want to do is hit the wall... If only there was a panacea that could produce permanent pain relief by using it once (in a gloomy voice)!" [26]* [Myeloma]<br>– *"Maybe [opioids] are gonna help you minimize it somewhat. But my pain stayed on 10...And I bring my pain pills with me because I don't want to feel the total 10 pain. But, waiting for it to subside, I'm just gonna be waiting forever…The pain won't go away." [17]* [Multiple myeloma]
Patient attitudes or concerns around opioids	9	[7, 13, 14, 17, 19-21, 26, 28]	– *"I take oral analgesics. Although the doctor persuades me to take medicines according to the prescribed dose, I secretly reduce the amount to only one tablet (oxycodone hydrochloride) per day for fear of addiction and side effects to the liver and platelets." [26]* [Lung cancer]

Unmet treatment need	Number of sources	Sources	Example supporting quotes
			– *"I had been a bit worried about the addiction problem with morphine but that's just a prejudice…It was just sheer prejudice because Ian Jury, the singer, used to, when he was singing, at concerts, take it all the time and I kind of knew him in London, when he was fighting his cancer but he was still singing, you know". [13]* [Pancreatic cancer]<br>– *"I don't want [opioids]. I've heard so many serious stories about people getting hooked on that stuff…remember Michael Jackson and Prince takin' all these strong meds? I didn't wanna deal with that." [17]* [Multiple myeloma]
Information and knowledge related to cancer treatment and pain	8	[3, 7, 8, 13, 14, 21-23]	– *"I believe the nervousness would have been less if I had heard from other patients that the calcium electroporation treatment is tolerable for pain." [22]* [Not reported]<br>– Author commentary*:* Overall, women believed they should be better informed prior to treatment that they might experience persistent pain as a side effect. Women felt being armed with this information in advance would have helped them manage their pain and prepare for the future. *[21]* [Breast cancer]<br>– Author commentary: Knowledge deficits with respect to the nature of breakthrough pain and medication usage among patients and health care workers hinder pain control management. *[14]* [Not reported]
Side effects of pain medication	7	[1, 9, 14, 17, 20, 26, 27]	– *"The first time they gave [opioids] to me, oh my God, that feeling is not good!...I was so high. And I told them, 'I don't never want that no more!'...I felt like the room was closing and I was flyin', it was ew!" [17]* [Chronic myeloid leukemia]<br>– *"[Opioids] make me kind of loopy and weird. Like really weird...I felt like a blank junky…You take one and you lay down and you wake up six hours later and take another one, just kind of a maintenance thing...It's just too, too heavy. Not enough motivation. Sleepy all the time." [17]* [Non-Hodgkin's lymphoma]<br>– *"Now… I completely lost my appetite, I would die from constipation and eating disorder if I continue to take these opioids.'' [14]* [Not reported]
Timely/easy access to pain medication	7	[8, 9, 11, 14, 15, 17, 28]	– *''The nurse did not seem to take my pain seriously. Sometimes it could take them almost half an hour to get my analgesic." [14]* [Not reported]<br>– Author commentary*:* In this regard, some participants stated that there are insufficient varieties of opioids in hospital wards and that they must wait a considerable amount of time to receive opioids. Therefore, patients must endure extreme pain for an extended period of time until the medication reaches the ward. *[11]* [Not reported]

Unmet treatment need	Number of sources	Sources	Example supporting quotes
			– Author commentary: When participants needed additional pain medication, they sometimes had to wait too long before it was administered, which made them feel vulnerable and unsafe. Some participants felt that they were not allowed to ask for extra medication and some participants expressed that they felt ignored by the nurse by having to wait too long for extra pain medication after the epidural pain treatment was stopped. *[8]* [Pancreatic cancer]
Lack of options in pain management	4	[11, 14, 18, 23]	– *"I think sometimes there's a naivete on the part of the physician and things become a little black-and-white for them. […] What I mean by that is sometimes people are not open to alternative approaches. That can be disheartening when they're not the ones that are suffering, but you are." [18]* [Lung cancer]
Access to healthcare professionals	3	[1, 15, 18]	– *"[I] haven't been to the GP for ages cause I just feel, well what can he do? We just keep phoning up for repeat prescriptions (…) None of the doctors down there have seen me for ages. If you said my name to them, they wouldn't even know what I looked like probably. I'm just a record to them. You know? I'm just a number in a way." [1]* [Breast cancer]

References

1. Adam R, de Bruin M, Burton CD, Bond CM, Clausen MG, Murchie P. What are the current challenges of managing cancer pain and could digital technologies help? BMJ supportive & palliative care. 2018;8(2):204-12.
2. Allsop MJ, Taylor S, Bennett MI, Bewick BM. Understanding patient requirements for technology systems that support pain management in palliative care services: a qualitative study. Health Informatics Journal. 2019;25(3):1105-15.
3. Appleyard SE, Clarke C. An interpretative phenomenological analysis of the experiences of older people self-managing cancer pain at home. Journal of Psychosocial Oncology. 2018;36(3):333-49.
4. Barrett L, Elliott E, Voorhaar M, Ingelgård A, Griebsch I, Wong B, et al. A mixed-methods study to better measure patient-reported Pain and fatigue in soft tissue sarcoma. Oncology and Therapy. 2023;11(1):129-43.
5. Benali K, Kebdani T, Hassouni K, El Kacemi H, El Majjaoui S, Benjaafar N. Experiences of Women Receiving Multifraction High Dose-Rate Brachytherapy for Cervical Cancer: A Prospective Qualitative Study. Journal of Cancer Therapy. 2022;13(6):311-22.
6. Cella D, Kelly K, de la Motte A, Toublan F, Pandya BJ, Shah MV, et al. The experience of patients with acute myeloid leukemia in remission post-transplant. Leukemia & Lymphoma. 2023:1-10.
7. Ekstedt M, Rustøen T. Factors that hinder and facilitate cancer patients' knowledge about pain management—a qualitative study. Journal of pain and symptom management. 2019;57(4):753-60. e1.
8. Englid MB, Jirwe M, Conte H. Perioperative Comfort and Discomfort: Transitioning From Epidural to Oral Pain Treatment After Pancreas Surgery: A Qualitative Study. Journal of PeriAnesthesia Nursing. 2023;38(3):414-20. e1.
9. Erol O, Unsar S, Yacan L, Pelin M, Kurt S, Erdogan B. Pain experiences of patients with advanced cancer: A qualitative descriptive study. European Journal of Oncology Nursing. 2018;33:28-34.

10. Everaars KE, Welbie M, Hummelink S, Tjin EP, de Laat EH, Ulrich DJ. The impact of scars on health-related quality of life after breast surgery: a qualitative exploration. Journal of Cancer Survivorship. 2021;15:224-33.
11. Hassankhani H, Hajaghazadeh M, Orujlu S. Patients' Experiences of Cancer Pain: A Descriptive Qualitative Study. Journal of Palliative Care. 2023;38(4):465-72.
12. Hodge FS, Line Itty T, Arbing RH, Samuel-Nakamura C. A window into pain: American Indian cancer survivors' drawings. Frontiers in Pain Research. 2022;3:1031347.
13. Koulouris A. The Epidemiology of Abdominal Pain in Inoperable Pancreatic Cancer and the Potential Role of Early Endoscopic Ultrasound-Guided Coeliac Plexus Neurolysis: University of East Anglia; 2021.
14. Liu Q, Gao L-L, Dai Y-L, Wang Y, Wang H-X, Luo X-J, et al. Breakthrough pain: a qualitative study of patients with advanced cancer in Northwest China. Pain Management Nursing. 2018;19(5):506-15.
15. Maly A, Singh N, Vallerand AH. Experiences of urban African Americans with cancer pain. Pain Management Nursing. 2018;19(1):72-8.
16. Martin S, Shah SN, Hepp Z, Harris N, Morgans AK. Qualitative analysis of pain in patients with locally advanced or metastatic urothelial carcinoma. Bladder Cancer. 2022;8(1):45-53.
17. Nabulsi NA, Nazari JL, Lee TA, Patel PR, Sweiss KI, Le T, et al. Perceptions of prescription opioids among marginalized patients with hematologic malignancies in the context of the opioid epidemic: a qualitative study. Journal of Cancer Survivorship. 2023:1-12.
18. O'Regan A, Fish LJ, Makarushka C, Somers T, Fitzgerald Jones K, Merlin JS, et al. Managing Chronic Pain in Cancer Survivorship: Communication Challenges and Opportunities as Described by Cancer Survivors. American Journal of Hospice and Palliative Medicine®. 2022.
19. Restivo L, Dudoit É, Duffaud F, Salas S, Dany L. "Fortunately I felt pain, or I would have thought I was on my way out": experiencing pain and negotiating analgesic treatment in the context of cancer. Journal of Psychosocial Oncology. 2023;41(2):150-65.
20. Schumacher KL, Clark VLP, Rabow MW, Paul SM, Miaskowski C. The experience of complex pain dynamics in oncology outpatients: a longitudinal qualitative analysis. Cancer nursing. 2021;44(2):136.
21. Smith MD, Manning J, Nielsen M, Hayes SC, Plinsinga ML, Coppieters MW. Exploring women's experiences with persistent pain and pain management following breast cancer treatment: A qualitative study. Frontiers in Pain Research. 2023;4:17.
22. Vestergaard K, Vissing M, Gehl J, Lindhardt CL. Qualitative investigation of experience and quality of life in patients treated with calcium electroporation for cutaneous metastases. Cancers. 2023;15(3):599.
23. Walsh EA, Chabria R, Vranceanu AM, Park ER, Post K, Peppercorn J, et al. Understanding pain related to adjuvant endocrine therapy after breast cancer: A qualitative report. European Journal of Cancer Care. 2022;31(6):e13723.
24. Wei X, Yu H, Dai W, Xu W, Yu Q, Pu Y, et al. Discrepancy in the perception of symptoms among patients and healthcare providers after lung cancer surgery. Supportive Care in Cancer. 2022;30(2):1169-79.
25. Whisenant MS, Srour SA, Williams LA, Subbiah I, Griffin D, Ponce D, et al. The unique symptom burden of patients receiving CAR T-cell therapy. Seminars in oncology nursing. 2021;37(6):151216.
26. Xu X, Cheng Q, Ou M, Li S, Xie C, Chen Y. Pain acceptance in cancer patients with chronic pain in Hunan, China: A qualitative study. International journal of nursing sciences. 2019;6(4):385-91.
27. Yeager KA, Quest TE, Vena C, Sterk CE. Living with symptoms: a qualitative study of black adults with advanced cancer living in poverty. Pain Management Nursing. 2018;19(1):34-45.
28. Yeager KA, Rosa WE, Belcher SM, Lee SM, Lee H, Bruner DW, et al. A Qualitative Study of the Pain Experience of Black Individuals With Cancer Taking Long-Acting Opioids. Cancer nursing. 2023:10.1097.
